# X-ray Fluorescence Uptake Measurement of Functionalized Gold Nanoparticles in Tumor Cell Microsamples

**DOI:** 10.3390/ijms22073691

**Published:** 2021-04-01

**Authors:** Oliver Schmutzler, Sebastian Graf, Nils Behm, Wael Y. Mansour, Florian Blumendorf, Theresa Staufer, Christian Körnig, Dina Salah, Yanan Kang, Jan N. Peters, Yang Liu, Neus Feliu, Wolfgang J. Parak, Anja Burkhardt, Elisabetta Gargioni, Sabrina Gennis, Sharah Chandralingam, Finn Höeg, Wolfgang Maison, Kai Rothkamm, Florian Schulz, Florian Grüner

**Affiliations:** 1 Fachbereich Physik, Universität Hamburg and Center for Free-Electron Laser Science (CFEL), Luruper Chaussee 149, 22761 Hamburg, Germany; oliver.schmutzler@desy.de (O.S.); florian@blumendorf.net (F.B.); theresa.staufer@desy.de (T.S.); ckoernig@mail.desy.de (C.K.); 2 Department of Chemistry, Universität Hamburg, Bundesstrasse 45, 20146 Hamburg, Germany; sebastian.graf@chemie.uni-hamburg.de (S.G.); sharah.chandralingam@chemie.uni-hamburg.de (S.C.); Finn.Hoeeg@gmx.de (F.H.); wolfgang.maison@chemie.uni-hamburg.de (W.M.); 3 Department of Radiotherapy and Radiation Oncology, University Medical Center Hamburg-Eppendorf, Martinistraße 52, 20246 Hamburg, Germany; n.behm@uke.de (N.B.); w.mansour@uke.de (W.Y.M.); dinasalah@sci.asu.edu.eg (D.S.); japeters@physnet.uni-hamburg.de (J.N.P.); e.gargioni@uke.de (E.G.); sabrina@gennis.info (S.G.); 4 Mildred Scheel Cancer Career Center, HaTriCS4, University Medical Center Hamburg-Eppendorf; Martinistraße 52, 20251 Hamburg, Germany; 5 Fachbereich Physik, Universität Hamburg and Center for Hybrid Nanostructures (CHyN), Luruper Chaussee 149, 22761 Hamburg, Germany; ykang@physnet.uni-hamburg.de (Y.K.); yangliu@desy.de (Y.L.); neus.feliu@physnet.uni-hamburg.de (N.F.); wolfgang.parak@uni-hamburg.de (W.J.P.); 6 Faculty of Science, Ain Shams University, Abbasiya, Cairo 11566, Egypt; 7 Fraunhofer Center for Applied Nanotechnology (CAN), Grindelallee 117, 20146 Hamburg, Germany; 8 Deutsches Elektronen-Synchrotron DESY, Photon Science, Notkestrasse 85, 22607 Hamburg, Germany; anja.burkhardt@desy.de

**Keywords:** XFI, X-ray fluorescence, nanoparticles, uptake, microsample

## Abstract

Quantitative cellular in vitro nanoparticle uptake measurements are possible with a large number of different techniques, however, all have their respective restrictions. Here, we demonstrate the application of synchrotron-based X-ray fluorescence imaging (XFI) on prostate tumor cells, which have internalized differently functionalized gold nanoparticles. Total nanoparticle uptake on the order of a few hundred picograms could be conveniently observed with microsamples consisting of only a few hundreds of cells. A comparison with mass spectroscopy quantification is provided, experimental results are both supported and sensitivity limits of this XFI approach extrapolated by Monte-Carlo simulations, yielding a minimum detectable nanoparticle mass of just 5 pg. This study demonstrates the high sensitivity level of XFI, allowing non-destructive uptake measurements with very small microsamples within just seconds of irradiation time.

## 1. Introduction

Nanoparticles have a wide range of potential medical applications [1]. For all potential in vivo applications, knowledge of the respective bio-distributions of the applied nanoparticle-containing systems is essential, e.g., for understanding targeting or long-term toxicity. While there are literally dozens of different image modalities available, many of them require particular properties of the nanoparticles to provide contrast, and not all of them are suited for imaging deep inside tissue. X-ray fluorescence imaging (XFI) has to offer interesting features in this direction.

XFI was used for quantitative element analysis first in 1928 [2]. Exciting heavy elements’ inner shell electrons from the K-or L-shell results in relaxation and element-specific X-ray emission (“X-ray echo”). High-Z elements produce hard X-ray photons [3] with high transmission capabilities, allowing for quantitative imaging of such tracer distributions in opaque objects. Since XFI is a non-destructive imaging modality, it explicitly allows for in vivo imaging of small animals [4,5,6], and it has recently been shown that medical XFI can even be applied to human-sized objects [7]. In-vitro single cell XFI studies with high resolution synchrotron scans achieved 2D sub-micron spatial resolution resolving cellular substructures and measured amounts of naturally occurring elements, such as Zn, Fe, Cu, Ni and others [8,9,10,11], or artificially introduced nanoparticles of gold or TiO_2_ [12,13,14].

The background in measured X-ray spectra is caused by (multiple) Compton scattering with corresponding energy loss of the incident photons, determining the minimal (local) amount of tracer material whose signal is statistically significant versus this background’s noise. Primary energy, target geometry, relative detector position, and solid angle covered by the detector heavily influence both the signal strength and the Compton background shape [7]. Typical sources for excitation are synchrotron radiation [15], X-ray tubes [16], or radioactive nuclides [17], while polarizing devices were also employed to achieve background reduction [18,19,20]. XFI is non-invasive, its spatial resolution is in principle only limited by the scanning beam size, thus allowing also for both in vivo and single cell measurements, with different levels of spatial resolution. While XFI was originally mainly used for investigating heavy-metal uptakes by workmen [21,22], today, it opens new possibilities in imaging and understanding pharmacokinetics [7].

Here, we present a pilot in vitro measurement of the uptake within a microsample consisting of only a few hundreds of cells. While the above mentioned single cell XFI allows for sub-cellular spatial resolution, the corresponding scanning time per cell is typically on the scale of hours. Therefore, the focus of our work is not on sub-cellular resolution XFI of a few single cells over some hours, but on measuring a microsample with up to about 100 cells within seconds to give the average uptake of functionalized gold nanoparticles (AuNPs) over the irradiated sample. This aim directly translates into the challenge of making a minimal local amount of AuNPs within the X-ray beam volume detectable. As a result, after numerical studies on the optimization of XFI, we found that this XFI approach could already work with just about 5 pg of total AuNP mass in the X-ray beam volume. This demonstrates the high sensitivity of XFI, which can only be achieved under optimal conditions, as discussed below. This mode of XFI operation scans an entire microsample of cells with just one stationary X-ray beam, reducing the irradiation time to just a few seconds.

As a demonstrator case for microsample XFI, prostate cancer cells (PC3 cell line) were exposed in vitro to gold nanoparticles (AuNPs) with different surface chemistries according to published protocols [23,24,25]. Different ligand coatings based on pegylated mercaptoundecanoic acid (PEGMUA), optionally modified with the prostate-specific membrane antigen (PSMA)-inhibitor (PEGMUA-PSMA-I), were attached to the AuNPs via thiol-gold chemistry. Details about the nanoparticle synthesis and characterization can be found in the Supplementary Materials together with a complementary uptake experiment, in which the uptake of AuNP was quantified with Inductively-Coupled Plasma Mass-Spectroscopy (ICP-MS).

## 2. Results

### 2.1. Synchrotron Based Experiment

For details on the cell and AuNP-preparation for the XFI-based uptake study, we refer to the Supplementary Materials. The cells to be studied were put into glass capillaries which were subsequently scanned with the X-ray beam from the DESY-synchrotron beamline P11. The scanning showed no differences in AuNP-masses, from which we have deduced a high degree of homogeneity of the loaded cells across the entire capillary.

**Figure 1:**
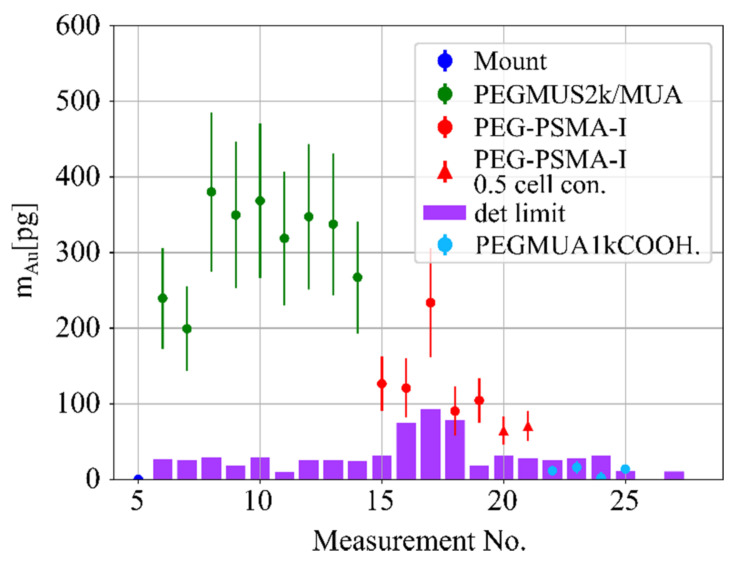
Reconstructed gold mass with corresponding detection limits (magenta) for four different probes, PEGMUA2k/MUA (green), PEG-PSMA-I (red dot), PEG-PSMA-I with half the number of cells in the beam (red triangle) and cells with PEGMUA1kCOOH nanoparticles (light blue dots). The nanoparticles were incubated at 12.5 nM with PC3 cells for 16 h, see Figure 2 The X-ray beam-sample-intersection volume, i.e., the volume of the sample which was interrogated by synchrotron radiation was 0.0889 mm^3^.

The uptake results are shown in Figure 1, where the highest gold uptake of 300 to 450 pg within the X-ray beam volume was found for the AuNPs coated with a **PEGMUA2k/MUA**, 100 to 150 pg in the case of the **PEG-PSMA-I** functionalization, with the sample containing half the cell concentration also showing significantly lower gold intake, and below detection limits in the case of the **PEGMUA1kCOOH**-coated AuNPs. Hereby, PC3 cells had been incubated for 16 h with AuNPs at 12.5 nM NP concentration, which is equivalent to a gold concentration of 0.13 mg/mL. For details of the cell culture procedures and concentration calculations, we refer to the Supplementary Materials. The observation that uptake is higher for **PEGMUA2k/MUA** than for the **PEG-PSMA-I** functionalized nanoparticles points to the fact that the results of uptake experiments have to be interpreted with care. The fact that no **PEGMUA1kCOOH** coated nanoparticles were found to be internalized can most likely be explained by the effect of PEG. However, the focus of the present manuscript is towards benchmarking the technical features of XFI for detecting AuNPs in cells, and a detailed discussion relating the uptake of AuNPs to their different physicochemical properties [26] would be out of the scope of this study. To put our work, however, into the context of standard NP uptake studies, complementary cell uptake experiments, including a full data set obtained with ICP-MS, are also discussed in the Supplementary Materials.

The measured photon counts in the signal energy region must exceed the background level by three or more standard deviations (σ) to be regarded as a statistically significant signal. This implies that a false-positive signal has a probability of less than 0.15%. For reasons of simplicity and comparability, this probability value is represented by confidence intervals given in standard deviations of the standard normal distribution σ, hence called a 3σ-significance.

### 2.2. Limits and Extrapolations

Here, we want to discuss how to determine the sensitivity limit of XFI for AuNP uptake measurements, that is, the minimum total AuNP mass inside the X-ray beam-sample-intersection volume when irradiation time scales are to be on the order of seconds. Fluorescence tracer detection limits increase with the square root of applied incident photons, see Appendix A. For a fixed experimental setup, Equation (1):

(1) $$Z/m=A\sqrt{I_{eff}}$$

describes the relationship between the statistical tracer signal significance, *Z* (in units of one standard deviation σ), per total irradiated tracer mass, *m*, in the X-ray beam volume, and *I_eff_*, the effective number of incident photons, which is the number of photons applied for one measurement corrected for detector dead time. The constant *A* is a measure for general experimental sensitivity since its value determines how many photons are required for irradiation to achieve a certain level of statistical signal significance for a fixed tracer mass *m* in the scanning beam volume. This constant highly depends on the experimental setup and might change drastically when incident energy, detector placement, phantom size, or tracer change. No fundamental detection limit can be determined when the irradiation time is scalable, therefore, limits are discussed per incident effective photon.

Experimental data in units of statistical significance over tracer mass (see Equation (1)) are presented in Figure 3 as a function of $\sqrt{I_{eff}}$. In particular, a single measurement taken with an incident energy of 20 keV is shown together with data recorded using 15 keV. The detector position remained fixed at an angle of 30° during the experiment (see Section 4), other more advantageous detector placement angles between 0° and 90° (see Figure 4) were simulated for the irradiations with 15 keV by using the Monte Carlo tool GEANT4 [27,28]. Due to horizontally polarized primary photons, placing the detector in the plane of polarization (i.e., in 90°) yields the highest sensitivities due to significant Compton background reduction.

Figure 3 shows the global range, including both, the gold L_α_ and L_β_ peak (left), and the L_α_ range (right), which uses this peak only for significance calculations.

Figure 3 also shows linear fits to the data, both for the 20 keV and the 15 keV experiments. Ideally, the experimental fit and the simulation for the 30° detector position should overlap, as they represent identical scenarios. Reasons for deviations here are beamline-intrinsic fluorescence, due to, e.g., tantalum or platinum (see Figure 4), and target fluorescence due to the presence of bromine or zinc (see Section 4.3), which are not modeled in the simulations.

Furthermore, an ideal overlap is not expected because the L-shell peaks are located on a mostly flat plateau, which is imitated artificially (see Section 4.4). An agreement between simulation and experimental data is still expected. On the other hand, the L_β_-line already experiences the flank of the Compton peak, hence, a flat plateau is not a perfect approximation. Thus, the background is probably underestimated in the simulations, which yield a higher sensitivity. These effects explain the differing behavior of simulations in Figure 3.

The sensitivity of this method, given by the slope, *A*, of the fits, increases when detector angles converge to 90° (see Table 1). As can be seen by comparing simulations results intrinsically, a change of the detector angular placement from 30° to 90° would increase the global sensitivity from 2.18·10^−**7**^ σ/pg/$\sqrt{I_{eff}}$ to 1.06·10^−6^ σ/pg/$\sqrt{I_{eff}}$, almost an order of magnitude.

An optimal setup at 90° would yield an efficiency of 1.06·10^−6^ σ/pg/$\sqrt{I_{eff}}$. An assumed detection limit of Z = 3 σ and 25 pg gold requires 1.28·10^10^ X-rays, while 5 pg gold need 3.2·10^11^ $I_{eff}$. The beamline delivers 3·10^12^ photons per second with pinhole applied, that is, about one order of magnitude more photons than needed. However, to achieve a reasonable detector deadtime, the incident flux needs to be attenuated, nevertheless, a signal with significance of Z = 3 σ can be achieved within 1 s in the case of 5 pg.

Homogeneous cell samples over 1 mm^2^ might be irradiated without pinhole with a beam crosssection with 0.5 mm radius compared to previous 0.1 mm, thus increasing the number of irradiated cells by 25 and decreasing the detectable tracer per cell by a factor 5.

## 3. Discussion

Even though synchrotron beamtimes are sparse, the preliminary results obtained in this work show that the non-destructive XFI-method is able to deliver similar results as retrieved from invasive methods like ICP-MS. Synchrotrons are the most brilliant X-ray sources, i.e., they deliver X-ray pencil beams with high flux and narrow bandwidth, allowing for high-sensitivity XFI [7]. However, their access is limited, as they are typically overbooked. To overcome these limits, our group also works on ultra-compact laser-driven Thomson X-ray sources [29], an approach envisaged for clinical use in the future. If such compact X-ray sources become available, one could also imagine using XFI directly at a hospital on fine-needle biopsies taken from a tissue. As XFI is non-destructive, the biopsy could still be used for routine pathology afterwards. The advantages of XFI are mainly two-fold. First, XFI leaves the samples unchanged, hence, it is a non-destructive method. The upper limit for the incident number of photons (and hence the resulting imaging sensitivity) is given by the irradiation-dose-dependent energy deposition, which is mainly transferred into a temperature rise. Secondly, XFI is quite sensitive, we can conclude that for the XFI-uptake assessment, only few cells are needed. As shown in the experimental part, it is possible to irradiate a cell microsample with just one stationary X-ray beam to get the uptake information within seconds.

As discussed above, a microsample containing a total amount of just 5 pg AuNPs is measurable within seconds under optimal conditions. However, to have a somewhat larger microsample for averaging over more cells, we conclude that few hundreds of cells suffice for an assay screening. The irradiation time can even further be reduced if more detectors are used at the same time or detectors with a larger area are available. Quantitatively, the amount of tracer in a microsample can be lowered by the square root of the number of detectors or the detection area increase, respectively. And since this is an in vitro technique, it scales simply with the square root of incident photons, which can be increased up to such high levels where the X-ray beam would deposit too much thermal heat—a situation that we have not reached in our experiments.

## 4. Materials and Methods

### 4.1. Cell Preparation and Particle Uptake

**Figure 2:**
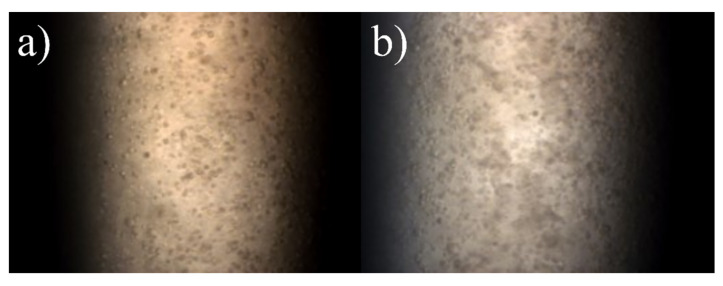
Microscopy image of a capillary with a cell concentration of (**a**) 0.5×10^7^ cells/mL, and (**b**) 1×10^7^ cells/mL.

**Figure 3:**
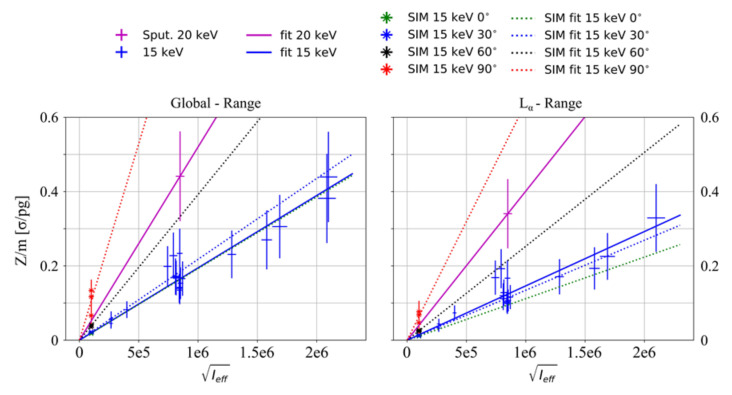
Significance[σ] per gold mass [pg] (i.e., total mass in beam-probe intersection) plotted over the root of effective incident photons for experimental and simulated data. Left: L_α_ and L_β_ gold fluorescence regions were considered. Right: only L_α_ on data is shown.

Cell cultivation was performed in 6-well-plates. After a 16 h exposure to a 12.5 nM concentration of 12 nm gold nanoparticles in medium, the cells were washed in phosphate buffered saline (PBS) and subsequently detached from well-plates by trypsin treatment. Cell counting took place before centrifugation, leaving pure cells which then were resuspended in a small volume of PBS, which was then mixed with 37 °C warm low melt agarose. This mixture was inserted into the capillaries, which were examined with XFI. This procedure led to a final cell concentration of 1·10^7^ cells/mL in the high concentration case and 0.5·10^7^ cells/mL in the low concentration case. Microscopy images of these samples are shown in Figure 2.

### 4.2. XFI Cell Uptake Measurements

Cells stored in a 45° tilted borosilicate glass capillary with 2 mm diameter and a beam diameter of 0.2 mm led to a beam target intersection volume of $\sqrt{2}$·π·(0.2 mm/2)^2^ ·2 mm ≈ 0.0889 mm^3^, with the factor $\sqrt{2}$ caused by the 45° tilt. Cell concentrations were set as 1 × 10^4^ cells/mm^3^ and 0.5 × 10^4^ cells/mm^3^. Assuming a homogeneous cell distribution in the capillary, the average cell numbers included in the beam target intersection were 888 or 444, respectively.

Estimating the volume filled by cells can be done with rough simplifications. Here, spherical cells with a diameter of 17.5 × 10^−6^ m are assumed, this shape can be motivated by Figure 2.

The cell volume percentage is calculated by multiplying the single cell volume with the cell concentration values, thus yielding about 1.57%, respectively 0.79% for half the concentration sample. Placing such spherical cells on a cubical grid with a distance of two cell diameters implies a lattice constant of three cell diameters. Such a distribution has a cell-filled volume of 1.94%, which meets the numbers above and is also is in agreement with the microscopy images shown in Figure 2.

### 4.3. Detector Setup

Scanning positions changed vertically to ensure different cells being probed per individual measurement. The Hitachi Vortex-EM detector was positioned at 30° from the vertical axis and at 6 cm distance from the probe-beam intersection (Figure 5). The P11 beamline at PETRA III is dedicated to macromolecular crystallography experiments, and the standard beamline setup did not allow for another angular detector placement [30]. Before the target, the beam is shaped by a 200 µm pinhole, and a few mm behind the target the beam is dumped. For later gold mass reconstruction, a 20.4 nm gold layer on a 1 mm thick silicon waver was used as a reference target.

The unattenuated photon flux at 15 keV at the sample position was determined by the beamline staff via diode measurement to be 3 × 10^12^ photons per second with pinhole applied [30]. The exposure time ranged between 30 and 1000 s while attenuators were applied to reduce the primary flux and thereby the detector dead time.

The energy region between 2 and 14 keV is prone to multiple background peaks, as light-element K-shell and heavy-element L-shell fluorescence occur in this region. To yield high sensitivity measurements, background caused by surrounding beamline components or the target itself needs to be addressed in background fit functions. To this purpose, a zero measurement without any target and a phantom measurement containing cells without treatment and agarose only were performed. The beamline intrinsic beamstop and pinhole had a visible impact on the gold L-shell region (energies between 8 and 13 keV), since they caused fluorescence of tantalum and platinum, whose peaks can be seen in Figure 6.

**Figure 4:**
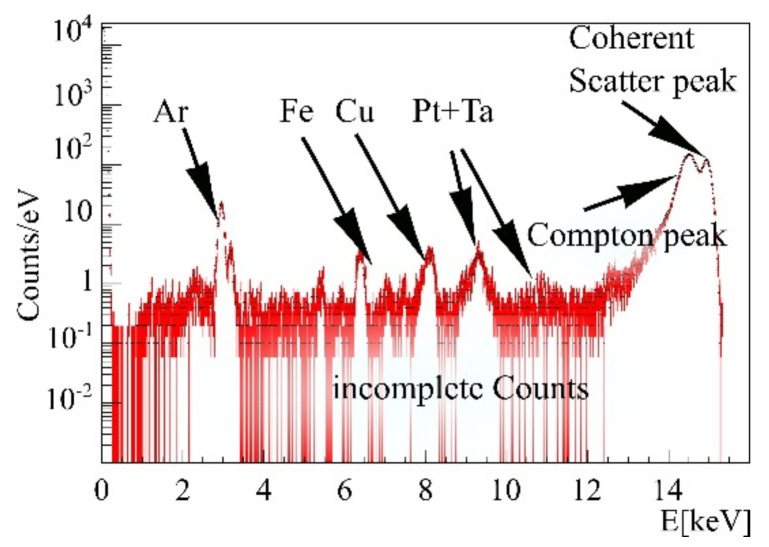
Measurement without sample. Beamline intrinsic fluorescence peaks are clearly visible.

**Figure 5:**
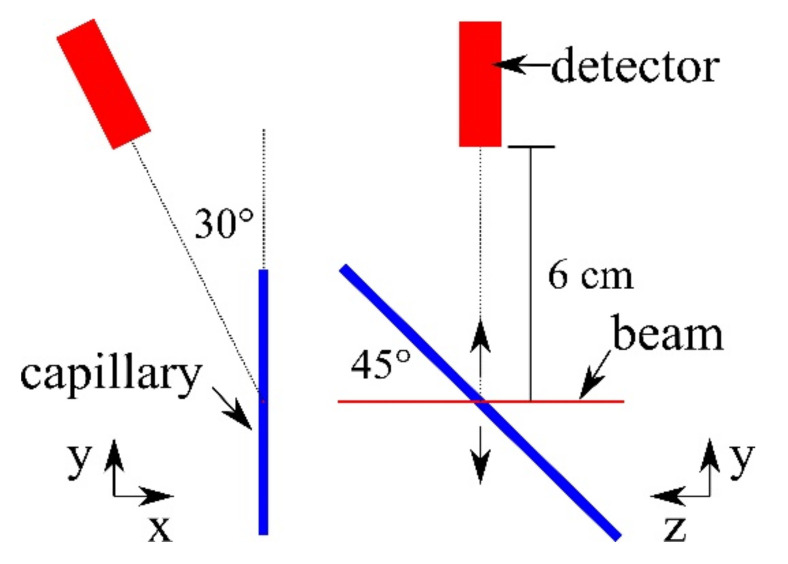
Schematic sketch of the capillary mount geometry. Left: frontal geometry, right: side view.

**Figure 6:**
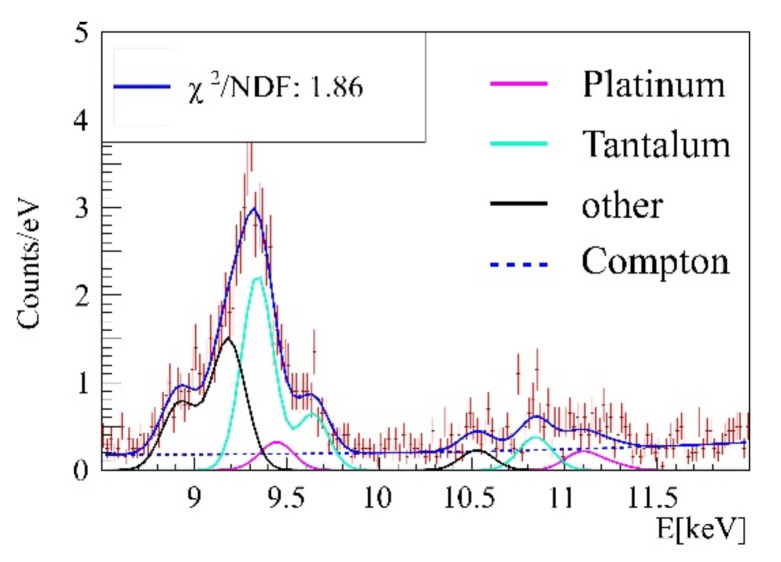
Background determination in the gold signal region, the main Ta (cyan) and Pt (pink) fluorescence lines are implemented accordingly to their intensities. Blue dotted: Compton background, black line: Other emission lines. Blue line: Fluorescence background for subsequent fits.

**Figure 7:**
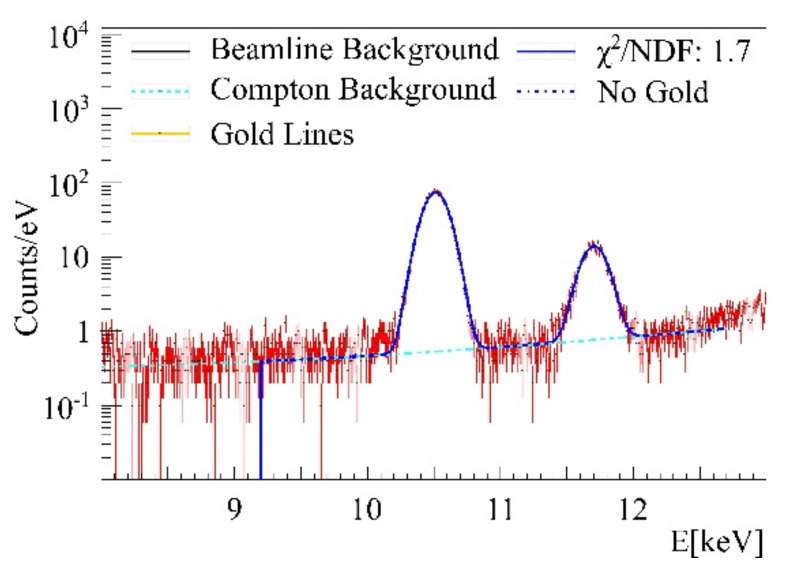
Capillary with agarose only, displaying two arsenic K_α_ lines at 10.5 keV and two K_β_ at 11.72 keV, each fitted with a single Gaussian.

**Figure 8:**
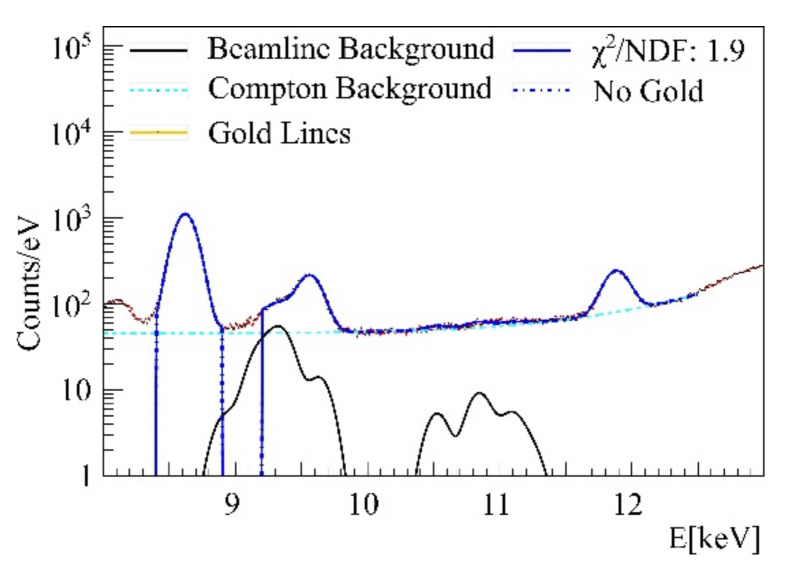
Cell mono layer showing zinc, below 9 keV and bromine above 12 keV.

Further, cell-probe intrinsic background was found in agarose containing arsenic and untreated cell samples showed peaks of bromine and zinc, as shown in the spectra in Figure 7 and Figure 8. Besides fluorescence background, scattered primary photons, mostly by Compton scattering, are causing the highest detector count rate and the first order Compton peak, consisting of singularly Compton scattered photons. Incomplete counts of such photons create a semi-constant plateau below this peak, shown in Figure 4. Even though minor compared to this peak, the plateau causes a major part of the gold L-shell fluorescence region background noise, diminishing fluorescence signal significance. Its height is proportional to first order Compton counts. A background function was therefore defined consisting of Compton background and fluorescence background peaks to distinguish between gold signal and other processes, as described below.

### 4.4. GEANT4 Simulation and Detector Background

Due to the small sample size, experimental verifications of the reported data by other modalities, such as ICP-MS, were limited. Plausibility and consistency are, therefore, demonstrated by Monte-Carlo simulations, using the GEANT4 tool with the polarized Livermore model for simulating photon interactions [27,28].

**Figure 9:**
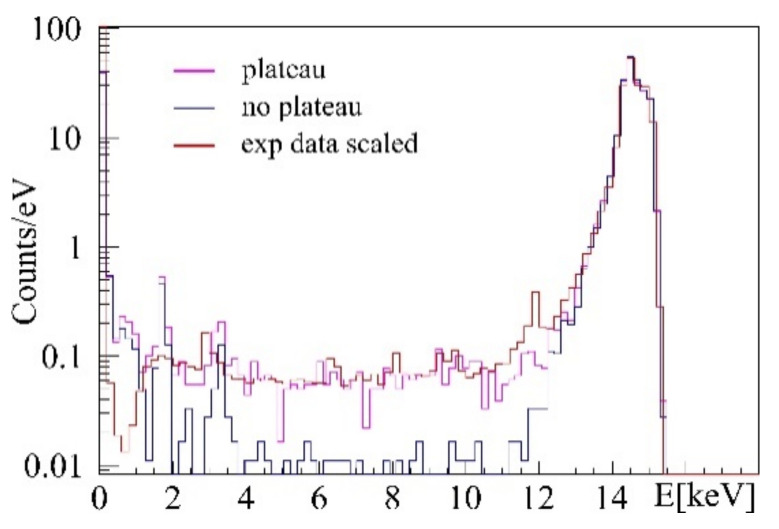
Comparison of simulated spectra with added plateau (magenta), no added plateau (blue), and an experimental spectrum (red).

In the L-shell fluorescence region, Compton scattering causes minor energy loss to incident photons, see Figure 9. If the fluorescence tracer energy is chosen below the Compton peak, the main background entries in the spectrum are caused by incompletely detected photons, creating a plateau reaching down to 1 keV. Incomplete counts in spectra do appear if photons interactions occur in the vicinity of detector chip surfaces. These events cause electron charge cloud leakage and a recording of energies smaller than the initial photon energy impinging on the detector.

GEANT4 simulations lacked a model for this effect, hence, the plateau was added post-simulation with its height being proportional to the number of photons in the main Compton peak. For each photon detected above 14 keV, an additional entry is created with a probability of 2.5% between 0.5 keV and the detected photon energy. A comparison between scaled experimental data and simulations with and without the added plateau is also shown in Figure 9. High fluxes at the synchrotron allow for sensitive measurements, however, meeting these high incident fluxes with GEANT4 simulations would increase the required simulation time to an unbearable amount. Therefore, scaling of experimental data is necessary to reduce needed computational resources and allow for comparison of experimental and simulated data.

Allowing for individualized setups without experimental restrictions, different detector placement angles were examined in simulations, as changes in Compton background counts and thus a change in the detector background plateau are expected.

**Figure 10:**
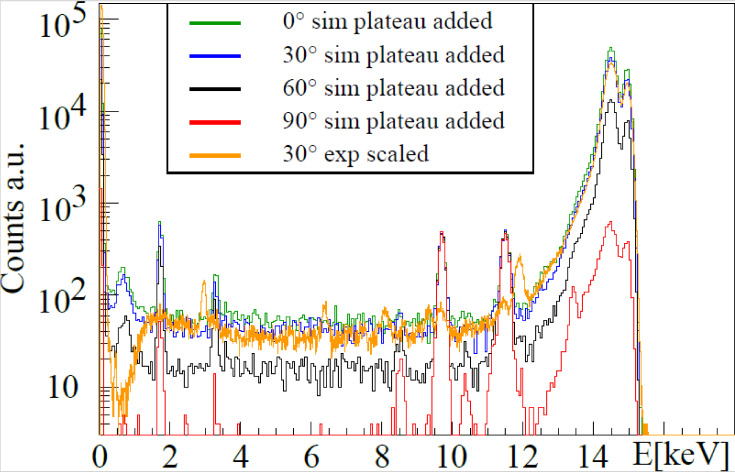
Simulated spectra with different detector angles, 0° (green), 30° (blue), 60° (black), and 90° (red). 90° equals a detector placement in the plane of polarization. The Au concentration here was 0.01 mg/mL. The detector angle in the experimental case was 30° for which an example spectrum is shown in yellow.

Angle variation spectra and scaled experimental spectra were compared, see Figure 10. Detector placement angles of 0°, 30°, 60°, and 90° were simulated where 90° means placing the detector in the plane of primary photon polarization, which reduces the background in the gold L-shell fluorescence region between 8 and 13 keV significantly. While a change to 60° does decrease the background by a factor of 2, almost two magnitudes in background reduction can be gained if optimal detector angles are chosen. A background decrease of two orders of magnitudes would increase the signal significance by a factor of 10. Furthermore, the overall count rate is similarly suppressed, reducing detector dead time and hence allowing for a smaller detector-target distance to reduce the overall measurement time.

### 4.5. Fits and Background Approximations

The tailing behavior of monoenergetic peaks in silicon detectors is described in [31]. Compton peaks in detected spectra lack monochromaticity, therefore, the equation was slightly altered to fit the left flank and low energy detector plateau with Equation (2):

(2) $$C\left(E\right)=p_{0}\left[1-\exp\left(-p_{1}\left(E-E_{0}\right)\right)\exp\left(p_{3}\left(E-E_{0}\right)\right)+\exp\left(p_{4}\left(E-E_{0}\right)+p_{5}\right)\right]$$

### 4.6. Mass Reconstruction

A thin sputter target with a 20.4 nm gold layer on a silicon wafer was used as a reference to determine unknown gold masses in cell probes. The variation of experimental conditions can cause variations of detector dead time, *τ*, target transmission factors of primary and signal photons, *T*, and measurement duration, *t*. Therefore, the gold mass, *m*, in cell targets can be determined as in Equation (3):

(3) $$m=m_{REF}\frac{F}{F_{REF}}\frac{\left(1-\tau_{REF}\right)}{\left(1-\tau \right)}\frac{\left(T_{REF}\right)}{T}\frac{\left(t_{REF}\right)}{t}$$

Here, *REF* variables were obtained during the reference measurement.

### 4.7. Statistics

Fluorescence signals are evaluated according to their significance *Z*, given in Equation (4), in units of one standard deviation (1*σ*):

(4) $$Z=\Phi^{-1}\left(1-p\right)$$

by applying the cumulative normal distribution function *Φ* with *p* value, as in Equation (5):

(5) $$p=\overset{\infty }{\underset{n⩾C_{s}+C_{B}}{\sum }}e^{-C_{b}}\frac{{\left(C_{B}\right)}^{n}}{n!}.$$

The significance threshold is set to *Z* = 3 here. *Z* scales linearly with the tracer mass *m*, (gold in this case), and the square root of the effective number of primary photons, *I_eff_*, as shown in Appendix A.

In Equation (6) the effective number of photons are related to photon flux *f*_0_, measurement duration *t*, primary beam attenuation *T_trans_*, and detector dead time *τ*:

(6) $$I_{eff}=\;f_{0}tT_{trans}\left(1-\tau \right).$$

As such, *I_eff_* is the number of photons, which can be effectively used for statistical calculations with the detector dead time taken into account.

## 5. Conclusions

Our pilot study on the uptake of functionalized AuNPs into prostate tumor cells shows that XFI is feasible for measuring a microsample containing a few hundred cells. While ICP-MS is already a widely used method, XFI has so far not been established yet for such microsample uptake studies, though it presents key advantages as it is a non-destructive method, leaving the sample unchanged, and at the same time provides high-sensitivity data. On the other hand, XFI requires brilliant X-ray beam sources for reaching such maximum sensitivity levels, which, in turn, only require microsamples of cells to retrieve the uptake data within seconds. While XFI is already used in single cell measurements [11] as well as for small-animal in-situ imaging [32], and is in principle even usable for human-sized objects [7], our present work closes the gap between single cell XFI, where the sub-cellular scanning of a single cell takes hours, and XFI for small-animals or large in-vitro cell samples. Since only microsamples are needed, one can now think of putting several different microsamples on top of each other inside a glass capillary for fast measurement of many such samples by simply moving the capillary through the X-ray beam. Since the cell preparation consists only of placing the cell microsamples into the capillary, a new type for high throughput uptake assay screening may come into reach.

## Figures and Tables

**Table 1:** Slopes of the fitted data, experimental and simulated, as shown in Figure 3. The upper row takes both fluorescence regions, L_α_ and L_β_, into account. The lower one only L_α_. By inserting the value of *A* in Equation (1), it is possible to extrapolate detectable gold masses per incident photon number.

Slope, *A*	0° sim	30° sim	60° sim	90° sim	30° exp
Global [σ/pg/$\sqrt{I_{eff}}$]	1.92·10^−7^	2.18·10^−7^	3.92·10^−7^	1.06·10^−6^	1.95·10^−7^
Lα [σ/pg/$\sqrt{I_{eff}}$]	1.12·10^−7^	1.35·10^−7^	2.53·10^−7^	6.39·10^−7^	1.46·10^−7^

## Data Availability

The data presented in this study are available on reasonable request.

## Supplementary Materials

Supplementary materials can be found at https://www.mdpi.com/article/10.3390/ijms22073691/s1.

## Abbreviations

XFI	X-ray Fluorescence Imaging
SNR	Signal to Noise Ratio
AuNP	Gold nanoparticle

## Appendix A

The figure of merit used for determination of fluorescence signals is the significance *Z* deduced from *p*-values *p_VAL_*. A similar useful measure is the signal to noise ratio, *SNR*. For extrapolation of sensitivities, a linear relation between *Z* and *SNR* assumed, the validity of this assumption is shown in the following.

The number of fluorescence photons *F* for significance calculation in a Gaussian peak with mean *μ*, RMS = *σ*, height *S* and histogram bin width Δ*E* is calculated in a ±3*σ* region, which can be estimated by integration over all numbers as in Equation (A1):

(A1) $$F=\frac{1}{\Delta E}\overset{E+3\sigma }{\underset{E-3\sigma }{\int }}Se^{\frac{-{\left(E-\mu \right)}^{2}}{2\sigma^{2}}}dE\approx \frac{1}{\Delta E}\overset{\infty }{\underset{-\infty }{\int }}Se^{\frac{-{\left(E-\mu \right)}^{2}}{2\sigma^{2}}}dE=\frac{S\sigma \sqrt{2\pi }}{\Delta E}$$

Similarly, the number of background photons *U* in the same ±3*σ* region is calculated in Equation (A2) with the mean background bin entry *B*:

(A2) $$U=\frac{6\sigma B}{\Delta E}.$$

The *p* value *p_VAL_*, given in Equation (A3), is the probability of the number of background photons *U* fluctuating to *F* + *U* or higher in a Poissonian distribution:

(A3) $$p_{val}=\overset{\infty }{\underset{k=\left(F+U\right)}{\sum }}e^{-U}\frac{U^{k}}{k!}.$$

With the central limit theorem applied in Equation (A4), a Gaussian can be assumed for the Poissonian distribution with: *σ*^2^ = *U* and *μ* = *U*, which yields:

(A4) $$p_{val}\approx \frac{1}{\sqrt{2\pi U}}\overset{\infty }{\underset{F+U}{\int }}e^{\frac{-{\left(x-U\right)}^{2}}{2U}}dx=\frac{1}{2}\left[1-erf(\frac{F}{\sqrt{2U}})\right]$$

By definition, *p_VAL_* and significance *Z* are linked as shown in Equation (A5):

(A5) $$p_{val}=\frac{1}{\sqrt{\left(2\pi \right)}}\overset{-Z}{\underset{-\infty }{\int }}e^{\frac{-x^{2}}{2}}dx=\frac{1}{2}\left[1-erf(\frac{Z}{\sqrt{\left(2\right)}})\right],$$

therefore, a linear relation, given in Equation (A6), between *Z* and *SNR* is reasonable to assume for data with sufficient counts:

(A6) $$Z=\sqrt{\left(\frac{\sigma }{\Delta E}\right)}2\pi \frac{S}{\sqrt{B}}=\sqrt{\left(\frac{\sigma }{\Delta E}\right)}2\pi SNR.$$

*SNR* and also *Z* scales with the square root of applied initial photons as signal height *S* and mean background height *B* do both scale linearly with the number of initial photons the target is irradiated with.
