# Supplementary material for: X-ray Fluorescence Uptake Measurement of Functionalized Gold Nanoparticles in Tumor Cell Microsamples
Source: Int J Mol Sci. 2021 Apr 1;22(7):3691. doi: 10.3390/ijms22073691 (PMC8037401; doi:10.3390/ijms22073691)
Supplement: Supplementary file 1 [file ijms-22-03691-s001.pdf]

International Journal of
Molecular Sciences

MDPI

Communication

Supplementary Materials: X-ray Fluorescence Uptake Measurement of Functionalized Gold Nanoparticles in Tumor Cell Microsamples

1. **Ligand syntheses**
2. **Particle synthesis**
3. **Particle characterization**
4. **Cell culture and ICP-MS particle uptake protocols**
5. **Discussion of ICP-MS uptake results**
6. **XFI significances and fit details**
7. **References**

1. Ligand syntheses

General. All solvents, reagents and starting materials were purchased by commercial sources (ABCR, Acros Organics, Macherey-Nagel, Merck, Roth, Sigma-Aldrich, VWR Chemicals) and used without further purification. All solvents used for purification were distilled (pentane, EtOAc) or purchased in HPLC grade (CH_3CN, EtOH). All dry solvents (DMF, CH_2Cl_2) were purchased from Acros Organics in molecular sieve grade. Commercially purchased trimethylamine was distilled and stored under nitrogen atmosphere on molecular sieve. Thin layer chromatography (TLC) was performed on silica gel aluminium sheets (Macherey-Nagel, DC Kieselgel ALugram® Xtra SIL G/UV_{254}, layer thickness 0.2 mm). UV-active compounds were detected by UV light (λ = 254 nm). Non-fluorescent compounds were stained with molybdophosphoric acid (10% in EtOH (w/w)). Flash column chromatography was performed on silica gel (Macherey-Nagel, 60-200 μm). Reversed phase column chromatography was performed on C18 ec silica gel (Mercherey-Nagel, 100-50, 40–63 μm). All reactions were performed under nitrogen atmosphere using dry solvents.

Equipment and Purification. NMR spectra were measured on Bruker Avance III HD
600 MHz (AVIII600), Bruker Avance I 500 MHz (AV500), Bruker Avance I 400 MHz
(AV4001) und Bruker FourierHD 300 MHz (F300UHH). Chemical shifts

were calibrated with signals of residual non-deuterated solvents. NMR numeration of synthesized compounds does not match with IUPAC nomenclature and only serves for NMR assignment. High-resolution mass spectrometry (HRMS) analysis was performed using Agilent 6224 ESI-TOF (110-3200 m/z). IR measurements were performed on FT/IR-4100 (Jasco). Elemental analysis was conducted on an EuroEA Elemental Analyzer a HEKAtech HAT oxygen analyzer (Fa. EuroVector/Hekatech). Automated purification steps on RP silica were carried out with a puriflash® 430 (Interchim).

The preparation of the immobilizable PSMA ligands **MUA-AHX-GPI**, **MUA-AHX-PSMA-I** and **MUA-AHX-Glu** was performed as shown in Figure S1.

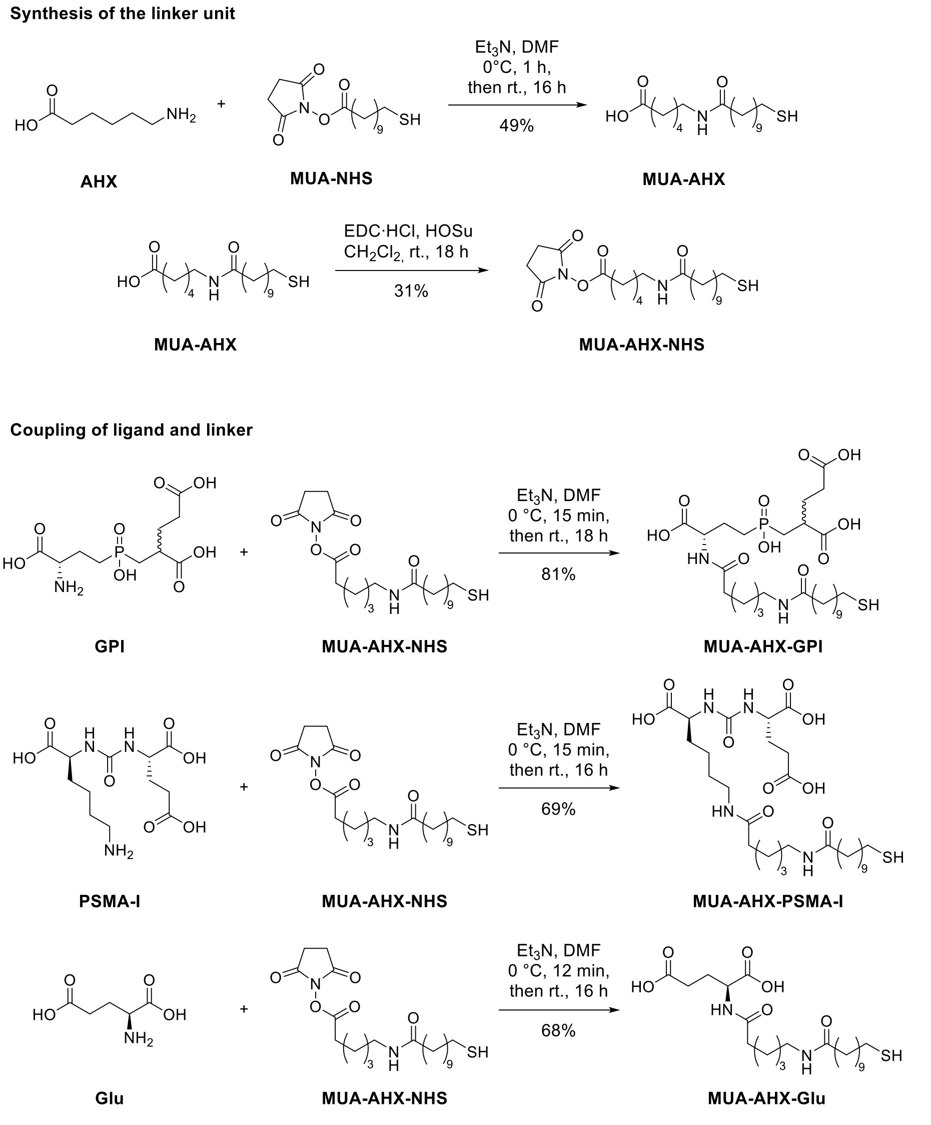

Figure S1. Reaction scheme for the preparation of **MUA-AHX-GPI**, **MUA-AHX-PSMA-I** and **MUA-AHX-Glu**.

GPI, **MUA-NHS** (and **PSMA-I**) were synthesized as described in literature [1–4]. Syntheses of the linker unit as well as the coupling of ligand and linker were performed using NHS-active ester chemistry. The detailed preparation of each compound together with corresponding analytical data is summarized below.

MUA-AHX:

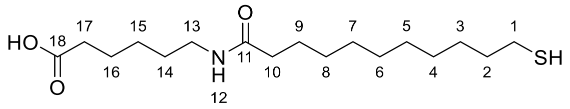

MUA-NHS (4.51 g, 14.3 mmol, 1.00 eq.) and **6-aminohexanoic acid** (**AHX**) (3.79 g, 28.9 mmol, 2.02 eq.) were separately dried under vacuum for 2.5 h. Subsequently, **AHX** was suspended in 75 mL of DMF and triethylamine (8.00 mL, 57.7 mmol, 4.04 eq.) was added. The cloudy suspension was cooled to 0 °C and a solution auf **MUA-NHS** in 50 mL DMF was added dropwise while stirring over a period of 1 h. Afterwards, the reaction mixture was stirred at room temperature for another 16 h. The solvent was removed under reduced pressure and the resulting colourless, waxy solid was purified by silica gel chromatography (pentane/EtOAc = 1.5:1 (+ 2% AcOH), v/v). To remove residues of acetic acid, the obtained solid was dissolved in 20 mL CH_2Cl_2 and coevaporated with toluene (2 x 15 mL). The product **MUA-AHX** (2.32 g, 7.00 mmol, 49%) was obtained as colourless solid.

Mp.: 77 – 80 °C.

$\boldsymbol{R_f}$**-value**: 0.30 (SiO_2, pentane/EtOAc = 1.5:1 (+ 2% AcOH), v/v), molybdophosphoric acid in EtOH (10 %)).

^{1}H-NMR (500.1 MHz, $CDCl_3$, 24.9 °C): δ [ppm] = 5.56 (br, s, 1 H, 12-H), 3.29 – 3.22 (m, 2 H, 13-H), 2.55 – 2.48 (m, 2 H, 1-H), 2.36 (t, $^3J_{(H,H)}$ = 7.3 Hz, 2 H, 17-H), 2.16 (t, $^3J_{(H,H)}$ = 7.6 Hz, 2 H, 10-H), 1.69 – 1.56 (m, 6 H, 16-H, 9-H, 2-H), 1.56 – 1.48 (m, 2 H, 14-H), 1.41 – 1.22 (m, 16 H, -CH_2, -SH).

Although the integral of the multiplet at 1.41 – 1.22 ppm is too high, indicating 16 instead of 15 protons, the distinct $^{13}C\{^1H\}$-NMR spectrum as well as the elemental analysis confirm the purity of the compound.

^{13}C{^{1}H}-NMR (100.6 MHz, $CDCl_3$, 21.9 °C): δ [ppm] = 178.4 (C-18), 173.7 (C-11), 39.4 (C-13), 37.0 (C-10), 34.2 (C-2), 33.9 (C-17), 29.6 (CH_2), 29.5 (CH_2), 29.44 (CH_2), 29.40 (CH_2), 29.38 (CH_2), 29.2 (C-14), 28.5 (CH_2), 26.4 (CH_2), 25.9 (C-9), 24.8 (C-1), 24.4 (C-16).

IR: $\tilde{\nu}$ [cm^{-1}] = 3307 (m), 2918 (s), 2850 (m), 1693 (s), 1631 (vs), 1534 (s), 1477 (m), 1470 (m), 1430 (w), 1417 (m), 1307 (w), 1286 (m), 1253 (m), 1204 (m), 939 (m), 729 (w), 718 (w), 682 (m), 581 (m).

HRMS-ESI (*m*/*z*): calc. for $C_{17}H_{34}NO_3S^+$ $[M+H]^+$: 332.2254, found: 332.2260.

C, H, N, S, O-analysis: calc. (%) for $C_{17}H_{33}NO_3S$: C 61.59, H 10.03, N 4.23, S 9.67, O 14.48; found: C 61.94, H 10.03, N 4.13, S 9.48, O 14.45.

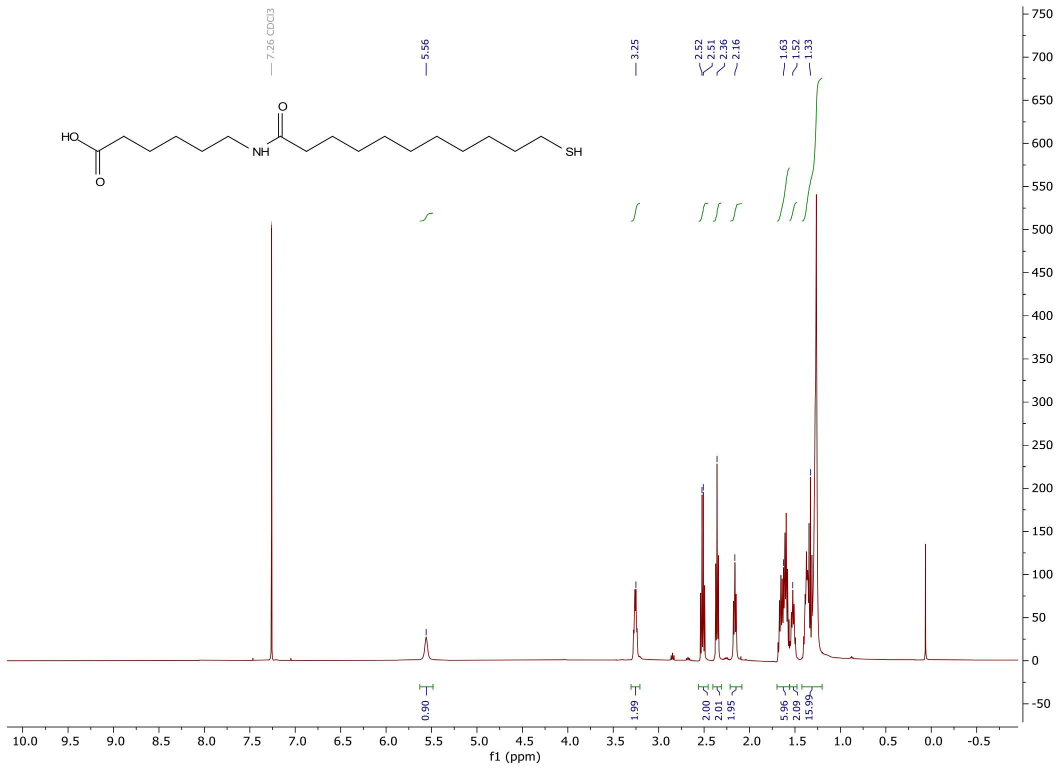

Figure S2. ^{1}H-NMR spectrum (500.1 MHz in $CDCl_3$) of **MUA-AHX**.

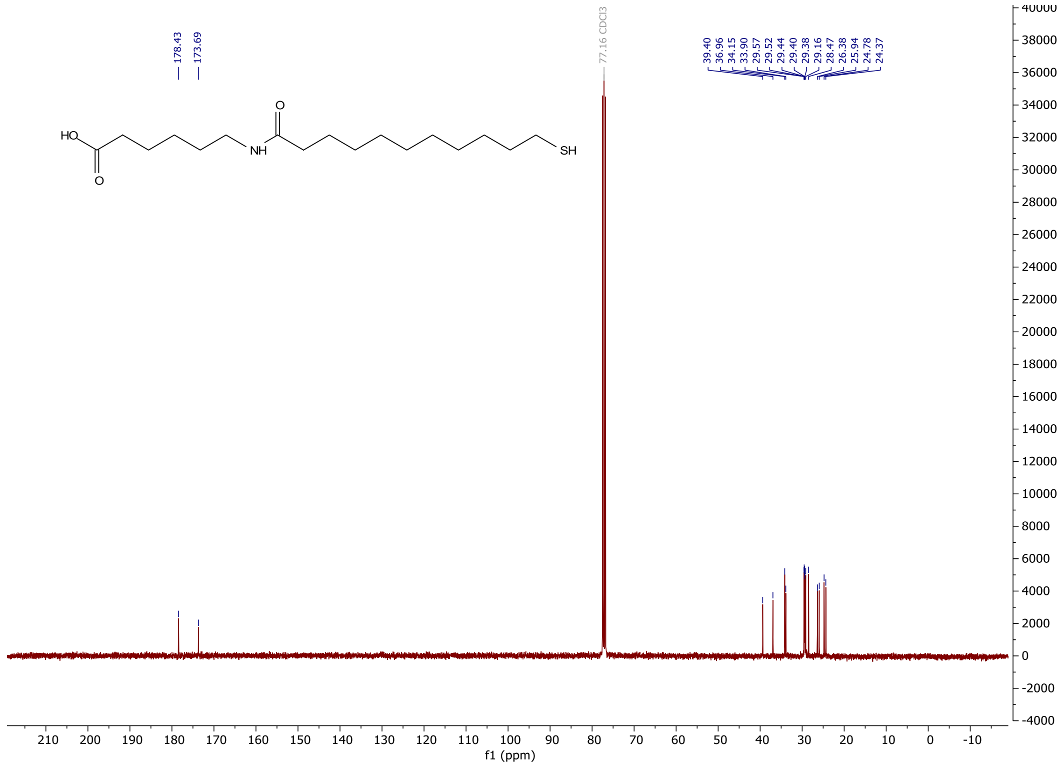

Figure S3. ^{13}C{^{1}H}-NMR spectrum (100.6 MHz in $CDCl_3$) of **MUA-AHX**.

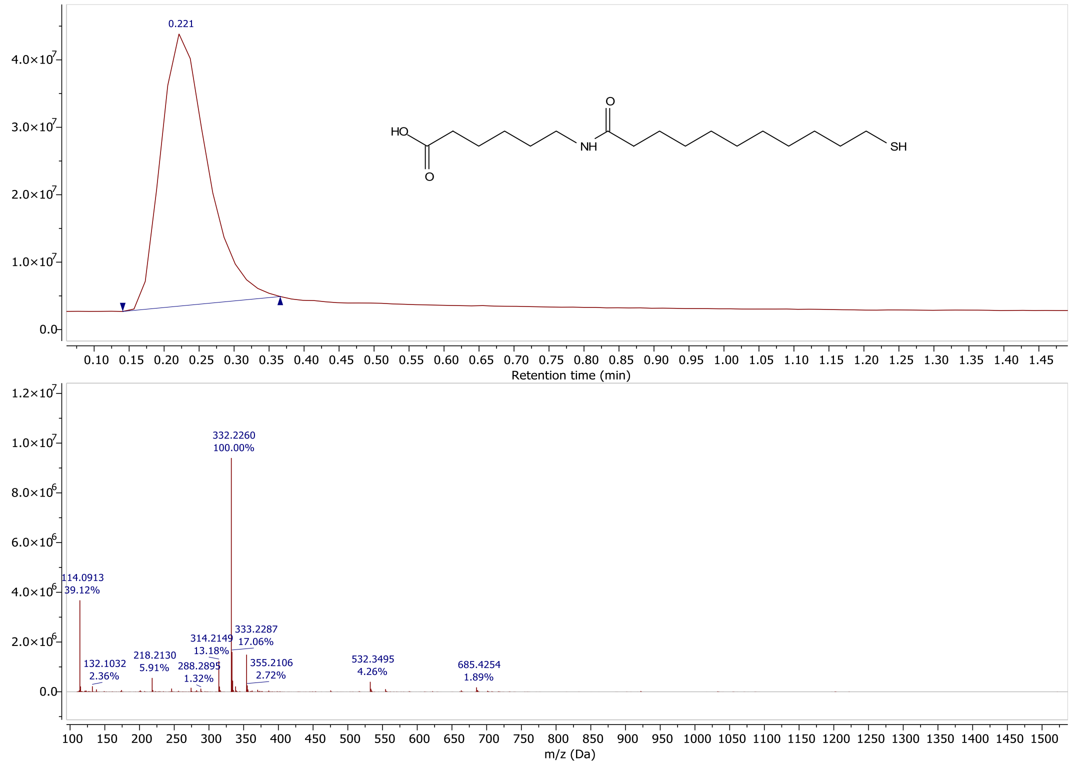

Figure S4. HRMS-ESI of **MUA-AHX**.

MUA-AHX-NHS:

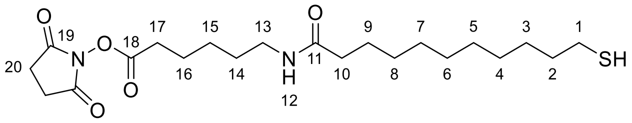

MUA-AHX (430 mg, 1.30 mmol, 1.00 eq.), *N*-hydroxysuccinimide (179 mg, 1.56 mmol, 1.20 eq.) and EDC·HCl (300 mg, 1.57 mmol, 1.21 eq.) were dried under vacuum for 2 h. The substances were dissolved in 60 mL CH_2Cl_2 and stirred at room temperature for 18 h. Subsequently, the colourless reaction mixture was washed with aq. NaCl solution (3 x 60 mL) and H_2O (1 x 60 mL). The organic layer was dried over Na_2SO_4, filtrated and concentrated under reduced pressure. The resulting colourless, solid residue was then purified by silica gel chromatography (elution with EtOAc). **MUA-AHX-NHS** (175 mg, 408 µmol, 31%) was obtained as colourless solid.

Mp.: 92 – 94 °C.

***R*$_f$-value**: 0.30 (SiO_2, EtOAc/pentane = 3:1 (v/v), molybdophosphoric acid in EtOH (10 %)).

^{1}H-NMR (500.1 MHz, $CDCl_3$, 21.9 °C): δ [ppm] = 5.65 – 5.58 (m, 1 H, 12-H), 3.29 – 3.23 (m, 2 H, 13-H), 2.89 – 2.79 (m, 4 H, 20-H), 2.62 (t, $^3J_{(H,H)}$ = 7.2 Hz, 2 H, 17-H), 2.54 – 2.48 (m, 2 H, 1-H), 2.18 – 2.12 (m, 2 H, 10-H), 1.82 – 1.74 (m, 2 H, 16-H), 1.66 – 1.50 (m, 6 H, 14-H, 2-H, 9-H), 1.50 – 1.41 (m, 2 H, 15-H), 1.40 – 1.22 (m, 13 H, -CH_2, -SH).

The multiplet at 1.50-1.66 ppm contains an overlap with the signal of water.

^{13}C{^{1}H}-NMR (100.6 MHz, $CDCl_3$, 21.9 °C): δ[ppm] = 173.5 (C-11), 169.3 (C-19), 168.6 (C-18), 39.1 (C-13), 36.9 (C-10), 34.2 (C-2), 31.0 (C-17), 29.6 (CH_2), 29.53 (CH_2), 29.46 (CH_2), 29.4 (CH_2), 29.2 (CH_2), 29.1 (CH_2), 28.5 (C-14), 25.94 (C-15/9), 25.91 (C-15/9), 25.7 (C-20), 24.8 (C-1), 24.4 (C-16).

IR: $\tilde{\nu}$[cm^{-1}] = 3332 (w), 2919 (m), 2848 (m), 1808 (w), 1790 (m), 1749 (vs), 1632 (vs), 1552 (s), 1462 (m), 1374 (w), 1359 (w), 1209 (vs), 1068 (vs), 1046 (m), 885 (m), 854 (w), 809 (w), 729 (w), 719 (w), 693 (w), 657 (m), 608 (w).

HRMS-ESI (*m*/*z*): calc. for $C_{21}H_{37}N_2O_5S^+$ [M+H]$^+$: 429.2418 found: 429.2425.

C, H, N, S, O-analysis: calc. (%) for $C_{21}H_{36}N_2O_5S$: C 58.85, H 8.47, N 6.54, S 7.48, O 18.66; found: C 59.07, H 8.53, N 6.61, S 7.43, O 18.61.

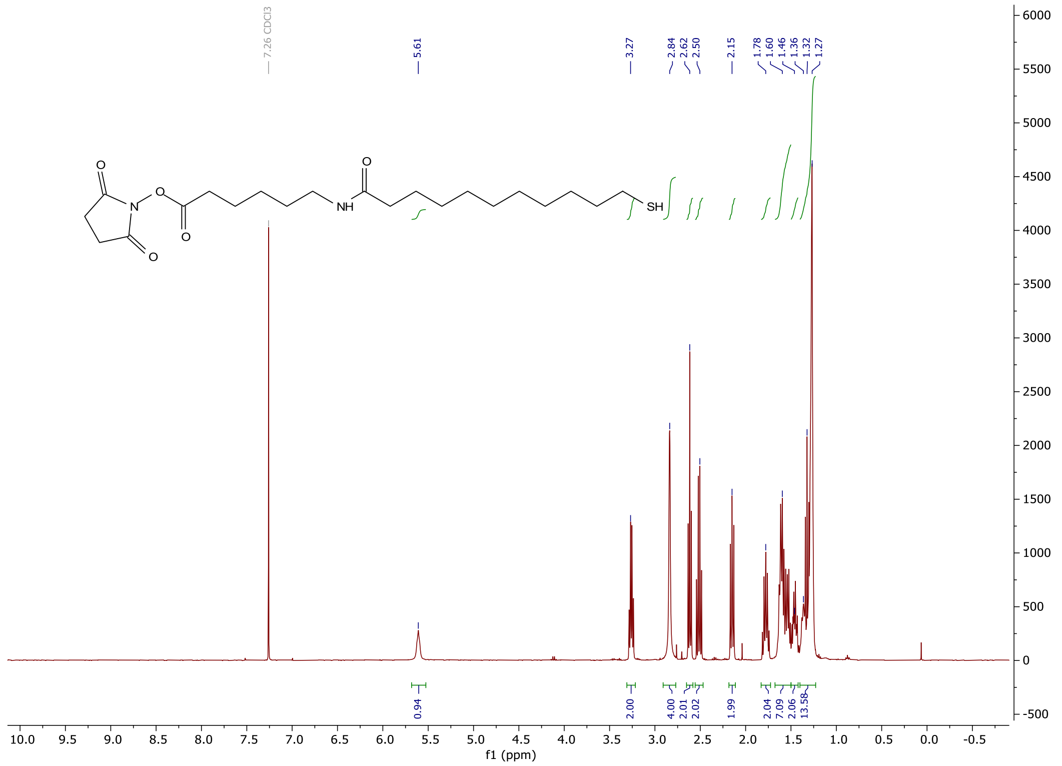

Figure S5. ^{1}H-NMR spectrum (500.1 MHz in $CDCl_3$) of **MUA-AHX-NHS**.

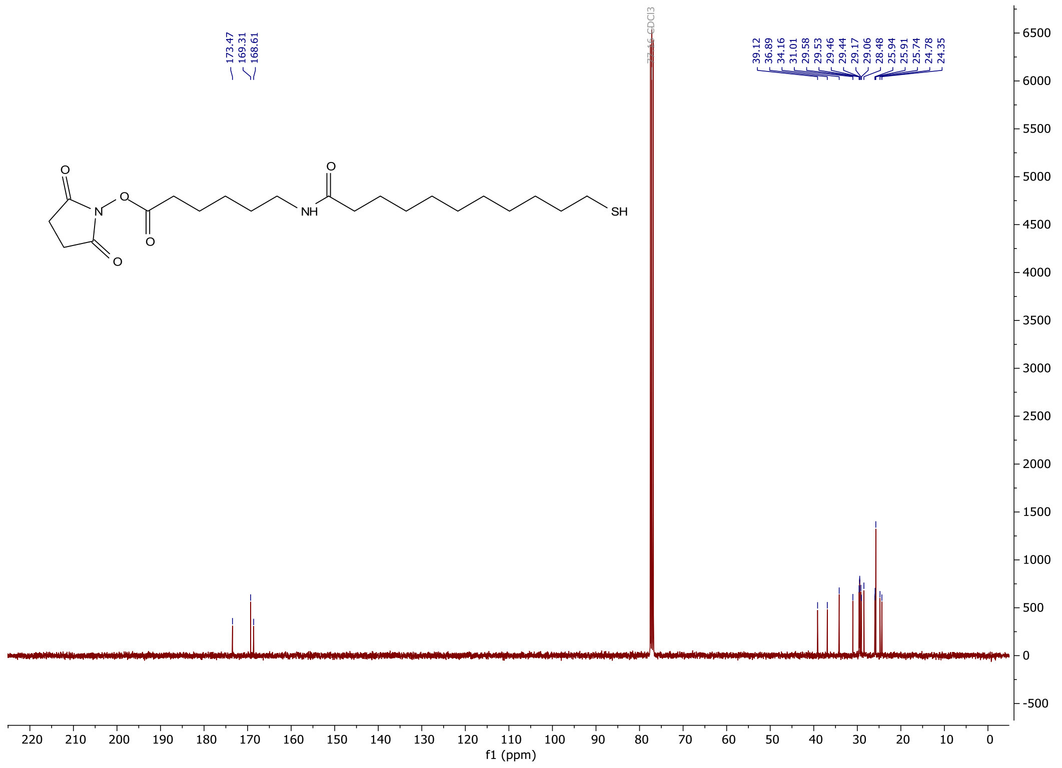

Figure S6. $^{13}C\{^{1}H\}$-NMR spectrum (100.6 MHz in $CDCl_3$) of **MUA-AHX-NHS**.

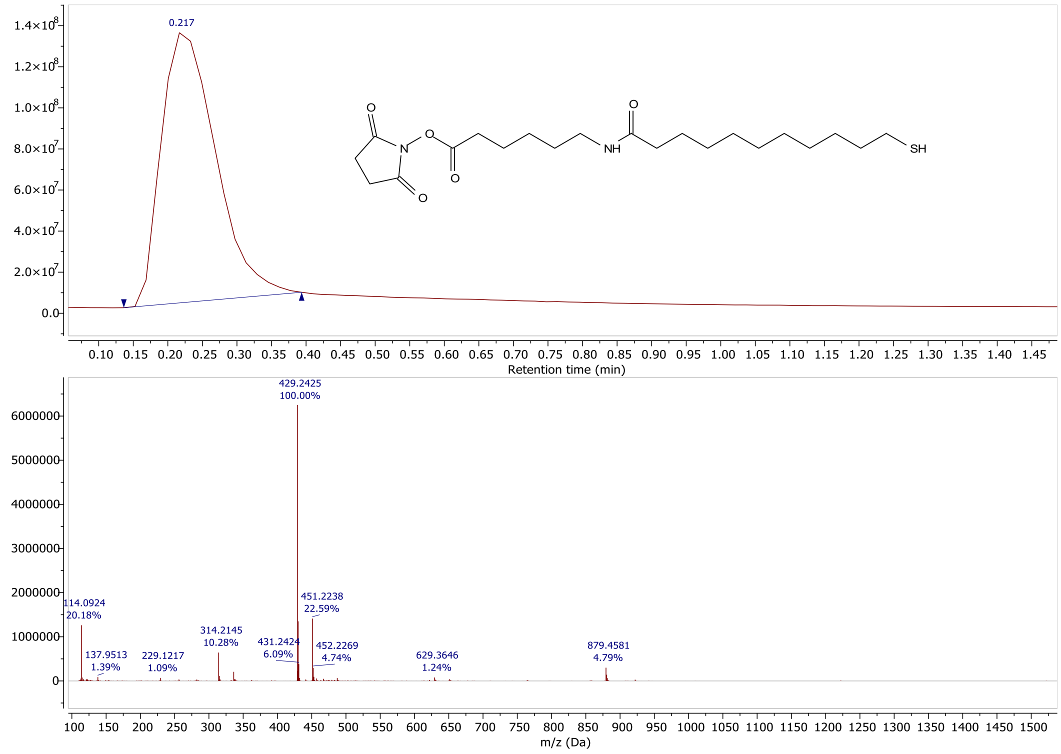

Figure S7. HRMS-ESI of **MUA-AHX-NHS**.

MUA-AHX-Glu:

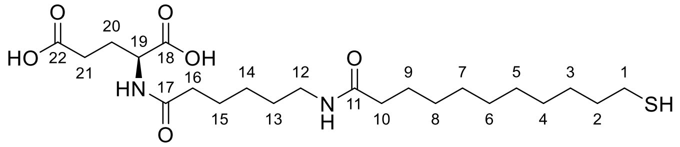

MUA-AHX-NHS (147 mg, 343 µmol, 1.00 eq.) and **L-glutamic acid** (**Glu**) (102 mg, 690 µmol, 2.01 eq.) were separately dried under vacuum for 2 h. Subsequently, the amino acid was suspended in 5 mL of DMF, cooled to 0 °C and triethylamine (0.29 mL, 2.1 mmol, 6.1 eq.) was added while stirring. To the cloudy suspension, a solution auf MUA-AHX-NHS in 6 mL DMF was added dropwise while stirring over a period of 12 min. Afterwards, the reaction mixture was stirred at room temperature for another 16 h. The solvent was removed under reduced pressure and the colourless, solid residue was purified by reversed phase silica gel chromatography (C18, H2O/ CH3CN = 98:2 → 0:100 (+ 0.01 % FA), (v/v), UV (254 nm)). The target molecule MUA-AHX-Glu (108 mg, 235 µmol, 68%) was obtained as colourless solid.

1H-NMR (400.1 MHz, CD3OD, 21.9 °C): δ [ppm] = 4.46 – 4.39 (m, 1 H, 19-H), 3.16 (t, 3J(H,H) = 7.0 Hz, 2 H, 12-H), 2.52 – 2.45 (m, 2 H, 1-H), 2.43 – 2.37 (m, 2 H, 21-H), 2.29 – 2.22 (m, 2 H, 16-H), 2.22 – 2.13 (m, 3 H, 20-Ha/b, 10-H), 1.99 – 1.87 (m, 1 H, 20-Ha/b), 1.69 – 1.47 (m, 8 H, 2-H, 13-H, 15-H, 9-H), 1.44 – 1.26 (m, 14 H, -CH2).

13C-DEPTQ-NMR (100.6 MHz, CD3OD, 21.9 °C): δ [ppm] = 176.29 (C-22/11/17), 176.25 (C-22/11/17), 175.0 (C-18), 53.0 (C-19), 40.2 (C-12), 37.2 (C-10), 36.6 (C-16), 35.2 (C-2), 31.3 (C-21), 30.60 (CH_2), 30.55 (CH_2), 30.4 (CH_2), 30.3 (CH_2), 30.2 (CH_2), 30.1 (C-13), 29.4 (C-14/3), 27.8 (CH_2), 27.5 (C-20), 27.1 (C-9), 26.5 (C-15), 25.0 (C-1).

IR: $\tilde{\nu}$ [cm^{-1}] = 3308 (w), 3070 (br, w), 2923 (m), 2850 (m), 2360 (w), 2343 (w), 1701 (br, m), 1633 (vs), 1541 (br, s), 1474 (w), 1460 (w), 1418 (w), 1254 (br, m), 1222 (w), 1211 (m), 1191 (br, m), 1130 (w), 942 (w), 730 (w), 684 (w), 597 (w).

HRMS-ESI (*m/z*): calc. for $C_{22}H_{41}N_2O_6S^+$ [M+H]$^+$: 461.2680 found: 461.2683.

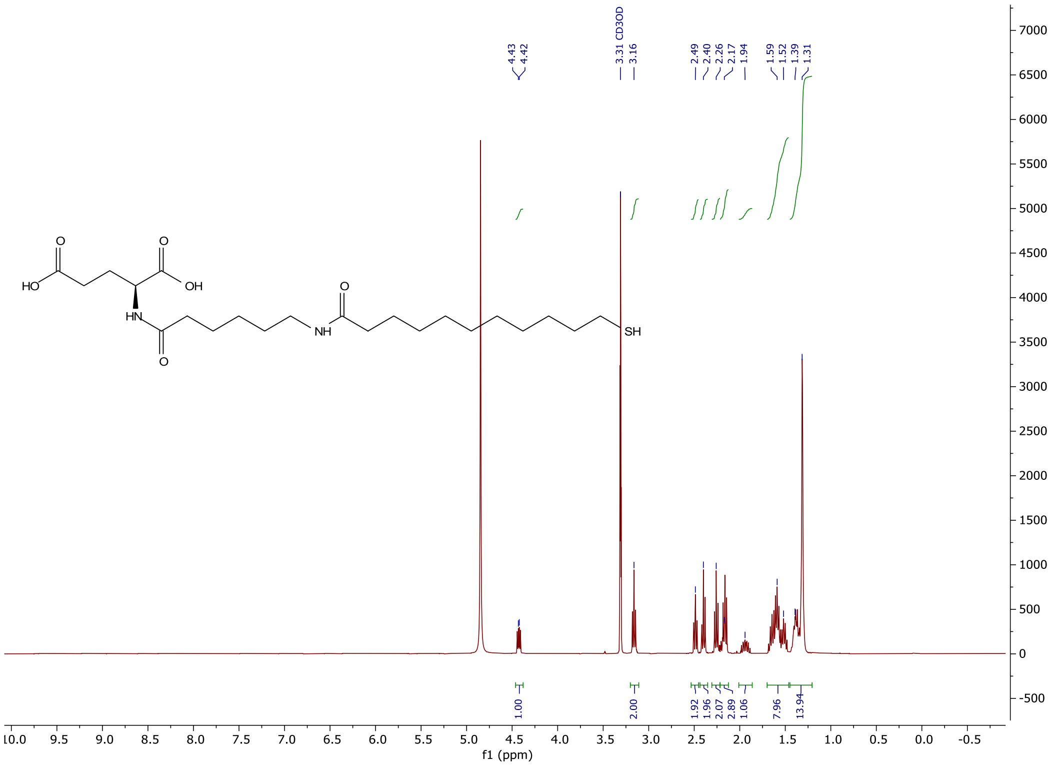

Figure S8. ^{1}H-NMR spectrum (400.1 MHz in CD_3OD) of **MUA-AHX-Glu**.

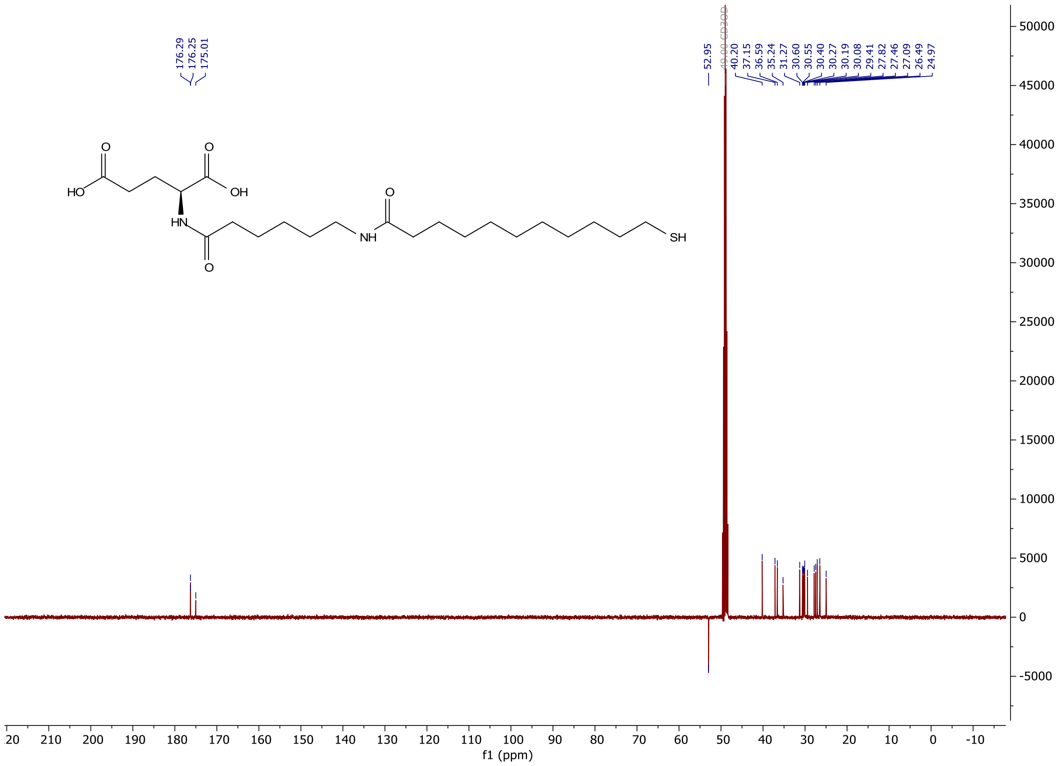

Figure S9. ^{13}C-DEPTQ-NMR spectrum (100.6 MHz in CD_3OD) of **MUA-AHX-Glu**.

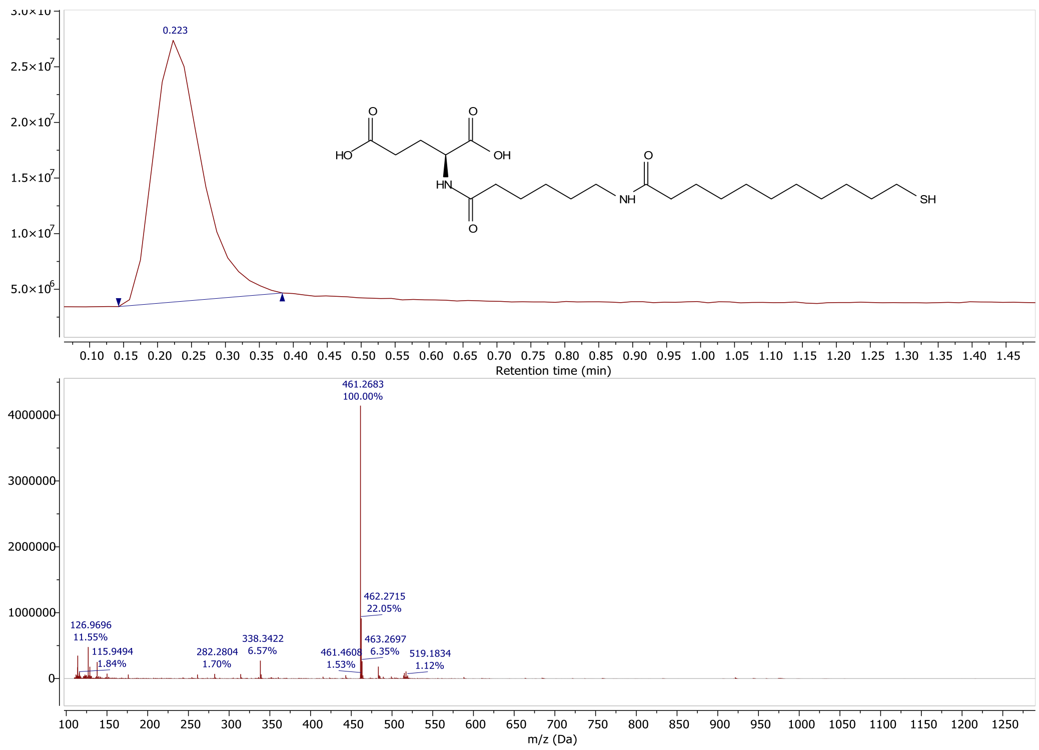

Figure S10. HRMS-ESI of **MUA-AHX-Glu**.

MUA-AHX-GPI:

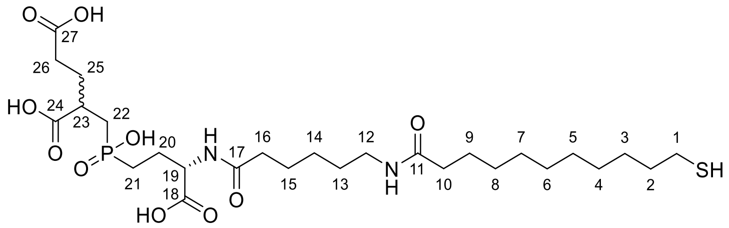

MUA-AHX-NHS (171 mg, 399 µmol, 1.00 eq.) and **GPI** (247 mg, 794 µmol, 1.99 eq.) were separately dried under oil pump vacuum for 4 h. Subsequently, **GPI** was suspended in 10 mL of DMF, cooled to 0 °C and triethylamine (0.58 mL, 4.2 mmol, 11 eq.) was added while stirring. To the cloudy suspension, a solution auf **MUA-AHX-NHS** in 7 mL DMF was added dropwise while stirring over a period of 15 min. Afterwards, the reaction mixture was stirred at room temperature for another 18 h. The solvent was removed under reduced pressure and the resulting colourless oil was purified by reversed phase silica gel chromatography (C_{18}, H_2O/CH_3CN = 98:2 → 0:100 (+ 0.01% FA), (v/v), UV (254 nm)). The target molecule **MUA-AHX-GPI** (202 mg, 323 µmol, 81%) was obtained as colourless solid.

^{1}H-NMR (600.1 MHz, CD_3OD, 24.9 °C): δ [ppm] = 4.46 – 4.41 (m, 1 H, 19-H), 3.16 (t, $^3J_{(H,H)}$ = 7.0 Hz, 2 H, 12-H), 2.83 – 2.75 (m, 1 H, 23-H), 2.51 – 2.46 (m, 2 H, 1-H), 2.44 – 2.32 (m, 2 H, 26-H), 2.28 (t, $^3J_{(H,H)}$ = 7.5 Hz, 2 H, 16-H), 2.26 – 2.19 (m, 1 H, 22-a/b), 2.19 – 2.11 (m, 3 H, 20-Ha/b, 10-H), 2.04 – 1.90 (m, 3 H, 20-Ha/b, 25-Ha+b), 1.89 – 1.75 (m, 3 H, 22-a/b, 21-H),

1.70 – 1.56 (m, 6 H, 2-H, 15-H, 9-H), 1.55 – 1.49 (m, 2 H, 13-H), 1.43 – 1.35 (m, 4 H, 14-H, 8-H), 1.35 – 1.28 (m, 10 H, -CH_2).

The ^{1}H-NMR spectrum shows an additional signal at 2.68 ppm (t, $^3J_{(H,H)}$ = 7.2 Hz, 0.23 H). This triplet corresponds to the -CH_2-S-S-CH_2- group of the disulfide product species. With an integral of the -CH_2-SH group of the target molecule of 1.77, the assumption can be made, that the corresponding disulfide of **MUA-AHX-GPI** was formed in a ratio of 1:15 in relation to the product. The appearance of the disulfide product can be caused by contact with air.

^{13}C-DEPTQ-NMR (150.9 MHz, CD_3OD, 24.9 °C): δ [ppm] = 177.63 (d, $^3J_{(P,C)}$ = 6.5 Hz, C-24a), 177.60 (d, $^3J_{(P,C)}$ = 6.5 Hz, C-24b), 176.40 (C-27a), 176.38 (C-27b), 176.3 (C-11+17), 174.5 (d, $^4J_{(P,C)}$ = 2.2 Hz, C-18, or 174.50 (C-18a), 174.48 (C-18b)), 53.9 (d, $^3J_{(P,C)}$ = 5.0 Hz, C-19a), 53.8 (d, $^3J_{(P,C)}$ = 5.0 Hz, C-19b), 40.2 (C-12), 39.9 (two overlapping doublets, C-23), 37.1 (C-10), 36.6 (C-16), 35.2 (C-2), 32.2 (C-26), 31.6 (d, $^1J_{(P,C)}$ = 92.4 Hz, C-22 or 32.0 (C-22a), 31.3 (C-22b)), 30.59 (CH_2), 30.55 (shoulder, CH_2), 30.4 (shoulder, CH_2), 30.3 (shoulder, CH_2), 30.2 (shoulder, CH_2), 30.1 (CH_2), 30.0 (d, $^3J_{(P,C)}$ = 6.0 Hz, 25a), 29.9 (d, $^3J_{(P,C)}$ = 6.0 Hz, 25b), 29.4 (C-14/8/3), 27.5 (C-14/8), 27.1 (C-9), 26.8 (d, $^1J_{(P,C)}$ = 89.2 Hz, C-21 or 27.1 (C-21a), 26.5 (C-21b)), 26.5 (C-15), 25.2 (d, $^2J_{(P,C)}$ = 2.9 Hz, C-20, or 25.16 (C-20a), 25.14 (C-20b)), 25.0 (C-1).

Because the product is a mixture of diastereomers, some carbon atoms give additional signals (labelled with a and b).

The presence of the disulfide dimer is supported by the appearance of a signal at 39.8 ppm (CH_2-S-S), which couples with the previously described triplet at 2.68 ppm (^{1}H-NMR) in the HSQC spectrum. It is further confirmed by the appearance of additional signals with low intensity from internal CH_2 groups which also appear as shoulder in the range of 30.6 – 30.2 ppm.

Other signals in the ^{13}C-DEPTQ-NMR spectrum: δ [ppm] = 39.8 (CH_2-S-S), 29.4 (**CH_2**-CH_2-S-S), 27.2 (CH_2-Disulfid), 26.6 (CH_2-Disulfid).

^{31}P{^{1}H}-NMR (162.0 MHz, CD_3OD, 26.5 °C): δ [ppm] = 51.21 (diastereomer 1), 51.17 (diastereomer 2).

IR: $\tilde{\nu}$ [cm^{-1}] = 3320 (w), 3096 (br, w), 2978 (w), 2920 (m), 2850 (m), 1703 (br, m), 1632 (s), 1555 (br, m), 1542 (br, m), 1461 (br, w), 1450 (br, w), 1415 (br, w), 1242 (br, m), 1169 (br, s), 1120 (br, m), 1017 (br, w), 957 (br, m), 781 (br, w), 731 (br, w), 718 (br, w).

HRMS-ESI (*m/z*): calc. for $C_{27}H_{50}N_2O_{10}PS^+$ [M+H]$^+$: 625.2919 found: 625.2928.

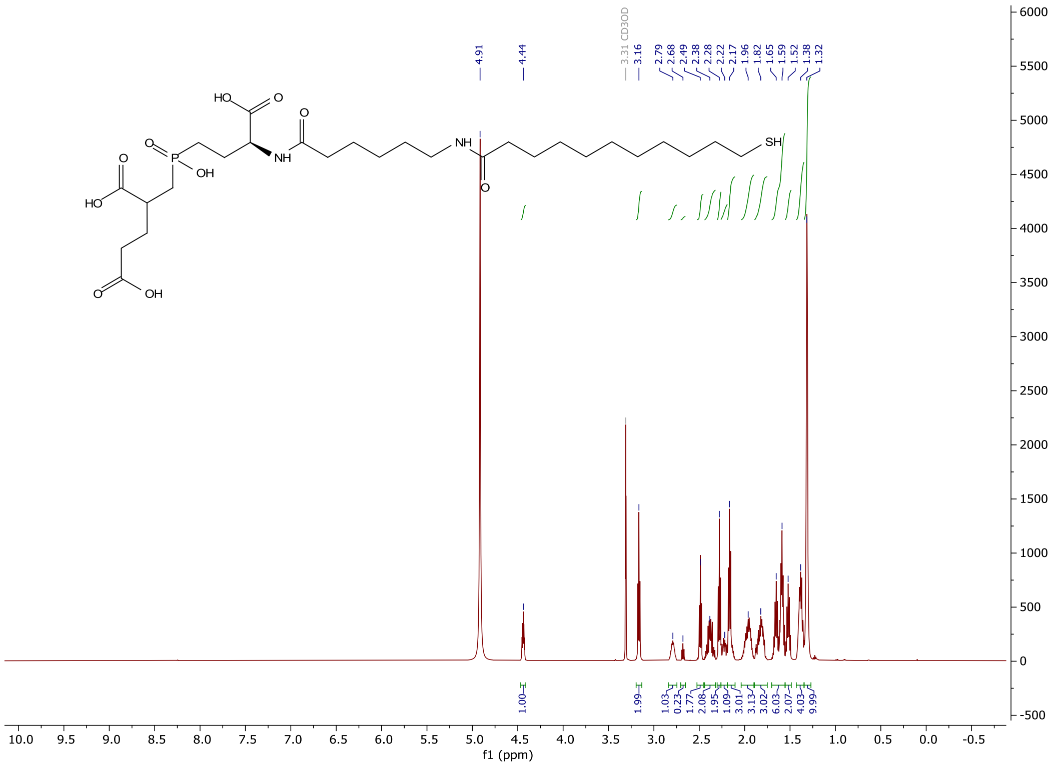

Figure S11. ^{1}H-NMR spectrum (600.1 MHz in CD3OD) of **MUA-AHX-GPI**.

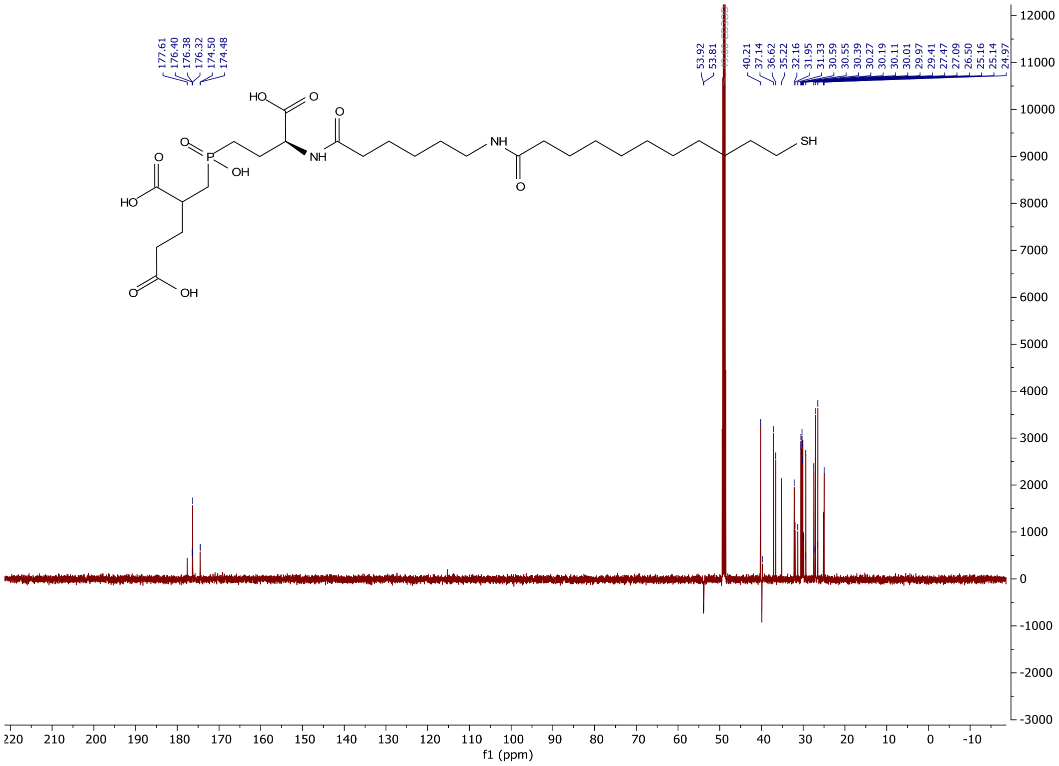

Figure S12. ^{13}C-DEPTQ-NMR spectrum (150.9 MHz in CD3OD) of **MUA-AHX-GPI**.

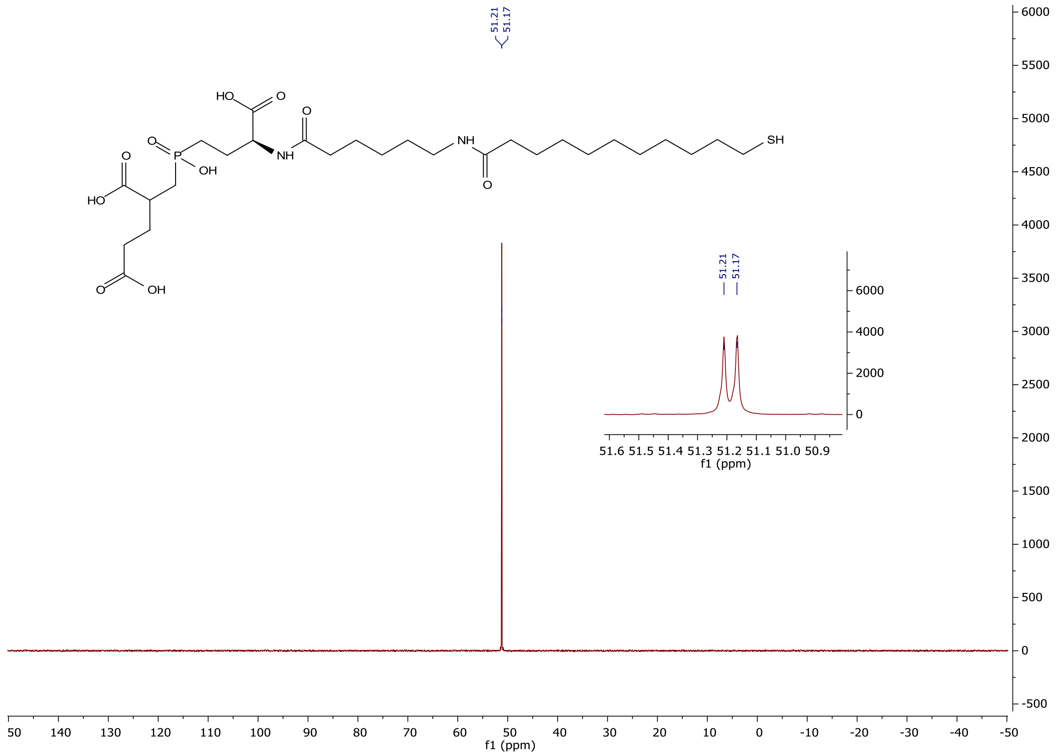

Figure S13. $^{31}P\{^{1}H\}$-NMR spectrum (162.0 MHz in CD_3OD) of **MUA-AHX-GPI**.

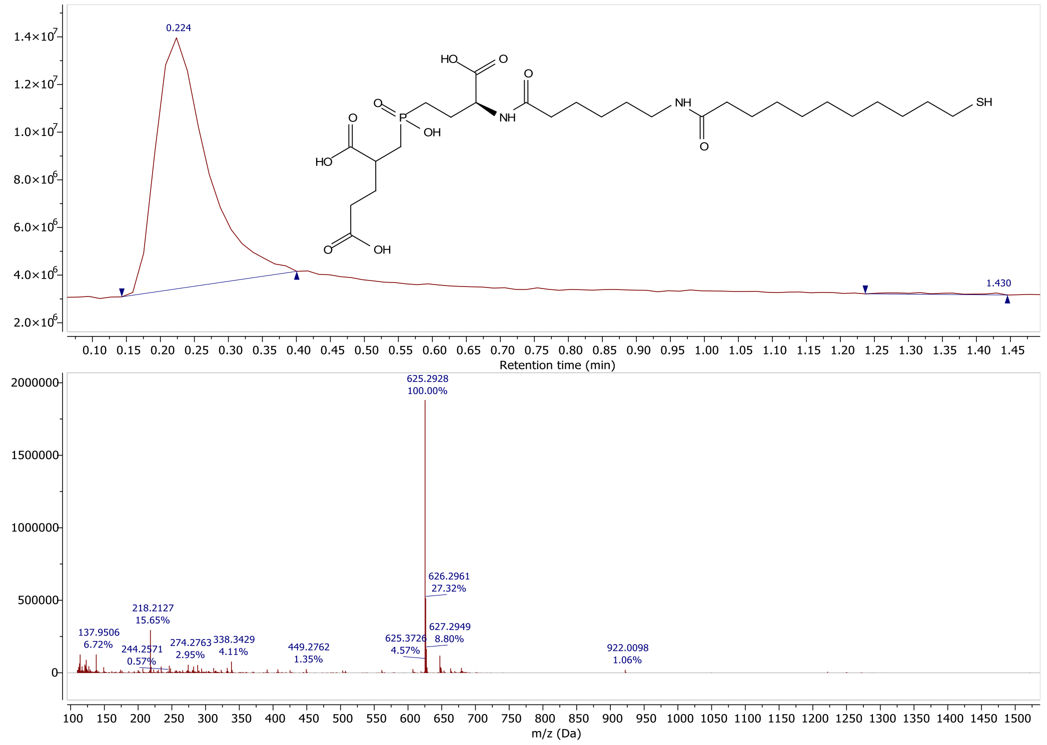

Figure S14. HRMS-ESI of **MUA-AHX-GPI**.

MUA-AHX-PSMA-I:

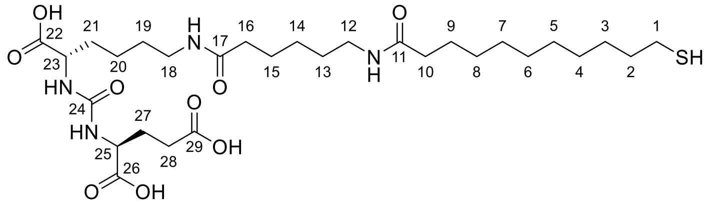

MUA-AHX-NHS (114 mg, 266 μmol, 1.00 eq.) and **PSMA-I** (126 mg, 395 μmol, 1.48 eq.) were separately dried under oil pump vacuum for 4 h. Subsequently, **PSMA-I** was suspended in 5 mL of DMF, cooled to 0 °C and triethylamine (0.25 mL, 1.8 mmol, 6.8 eq.) was added while stirring. To the cloudy suspension, a solution auf **MUA-AHX-NHS** in 6 mL DMF was added dropwise while stirring over a period of 15 min. Afterwards, the reaction mixture was stirred at room temperature for another 16 h. The solvent was removed under reduced pressure and the resulting colourless oil was purified by reversed phase silica gel chromatography (C_{18}, H_2O/ CH_3CN = 98:2 → 0:100 (+ 0.01% FA), (v/v), UV (254 nm)). The target molecule **MUA-AHX-PSMA-I** (116 mg, 183 μmol, 69%) was obtained as colourless solid.

^{1}H-NMR (600.1 MHz, CD_3OD, 24.9 °C): δ [ppm] = 4.33 – 4.29 (m, 1 H, 25-H), 4.28 – 4.24 (m, 1 H, 23-H), 3.21 – 3.12 (m, 4 H, 12-H, 18-H), 2.52 – 2.45 (m, 2 H, 1-H), 2.45 – 2.36 (m, 2 H, 28-H), 2.20 – 2.11 (m, 5 H, 27-Ha/b, 16-H, 10-H), 1.93 – 1.80 (m, 2 H, 27-Ha/b, 21-Ha/b), 1.70 – 1.56 (m, 7 H, 21-Ha/b, 2-H, 9-H, 15-H), 1.56 – 1.47 (m, 4 H, 19-H, 13-H), 1.46 – 1.37 (m, 4 H, 20-H, 14-H), 1.37 – 1.27 (m, 12 H, -CH_2).

^{13}C-DEPTQ-NMR (150.9 MHz, CD_3OD, 24.9 °C): δ [ppm] = 176.44 (C-29/22), 176.39 (C-29/22), 176.3 (C-11/17), 176.1 (C-11/17), 175.8 (C-26), 160.1 (C-24), 54.0 (C-23), 53.5 (C-25), 40.2 (C-12/18), 40.1 (C-12/18), 37.2 (C-16/10), 37.0 (C-16/10), 35.2 (C-2), 33.2 (C-21), 31.1 (C-28), 30.60 (CH_2), 30.55 (CH_2), 30.4 (CH_2), 30.3 (CH_2), 30.2 (CH_2), 30.1 (CH_2), 29.9 (CH_2), 29.4 (C-14/3), 28.9 (C-27), 27.5 (CH_2), 27.1 (C-9), 26.7 (C-15), 25.0 (C-1), 24.0 (C-20).

IR: $\tilde{\nu}$ [cm^{-1}] = 3308 (w), 3107 (br, w), 2978 (w), 2921 (m), 2851 (w), 2360 (vs), 2341 (vs), 1699 (br, m), 1633 (br, s), 1557 (s), 1458 (br, w), 1417 (br, w), 1252 (br, m), 1181 (br, m), 1128 (br, w), 669 (m).

HRMS-ESI (*m*/*z*): calc. For $C_{29}H_{53}N_4O_9S^+$ [M+H]$^+$: 633.3528 found: 633.3531.

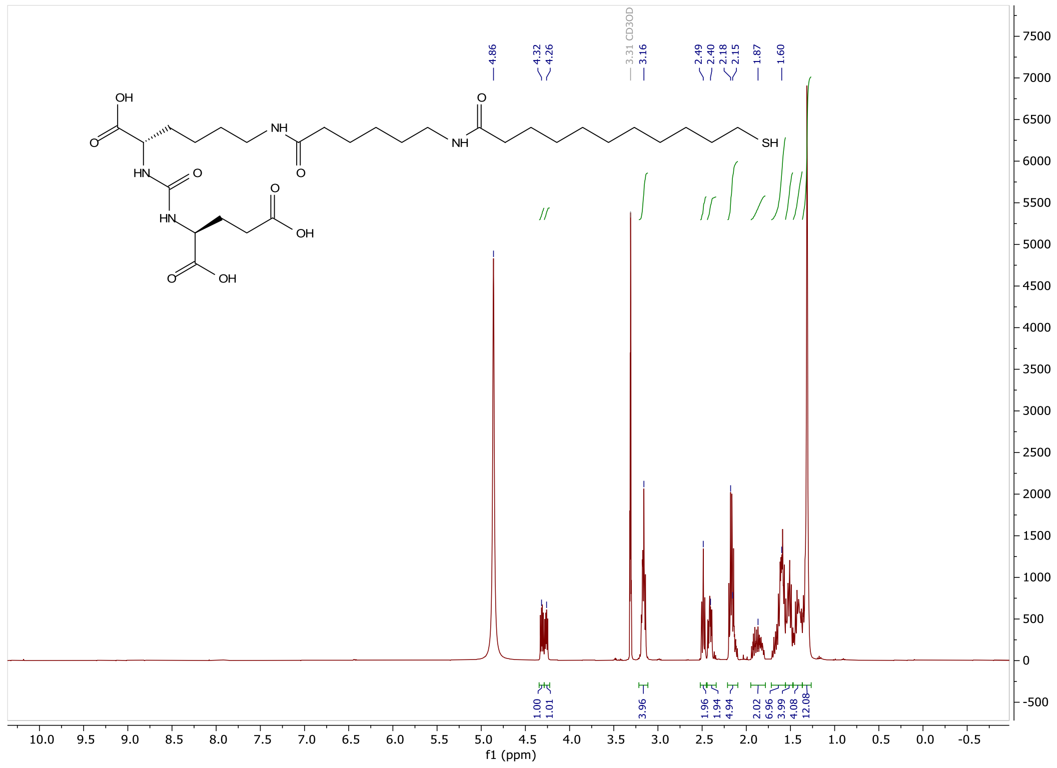

Figure S15. ^{1}H-NMR spectrum (600.1 MHz in CD_3OD) of **MUA-AHX-PSMA-I**.

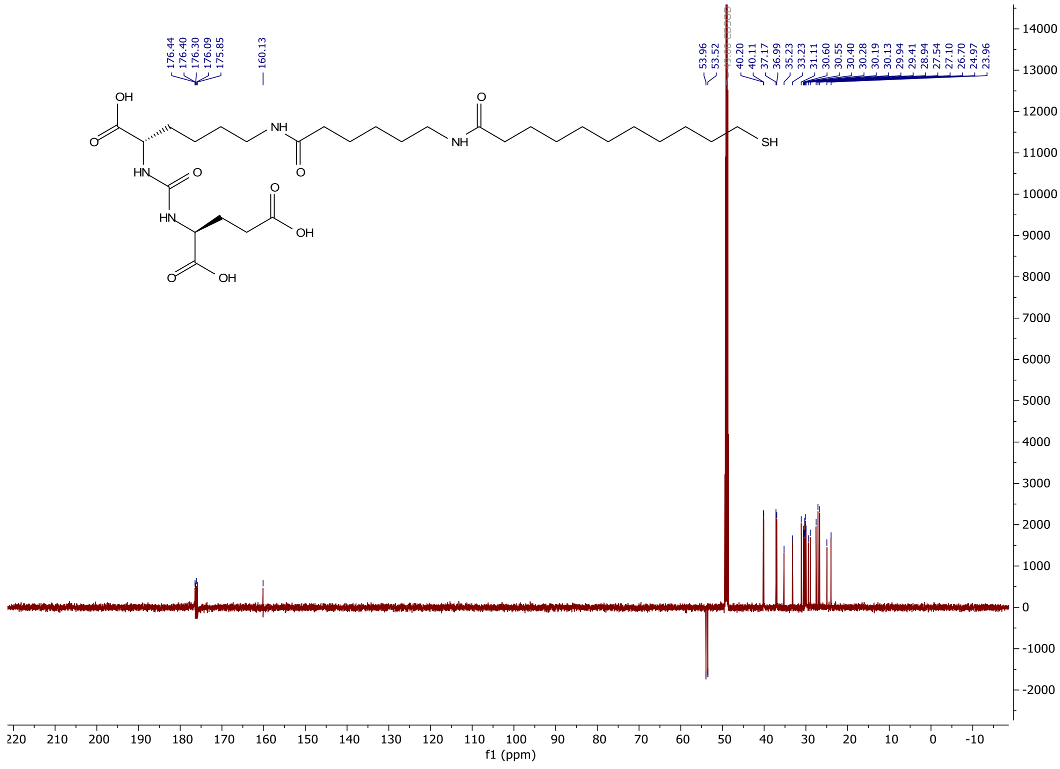

Figure S16. ^{13}C-DEPTQ-NMR spectrum (150.9 MHz in CD_3OD) of **MUA-AHX-PSMA-I**.

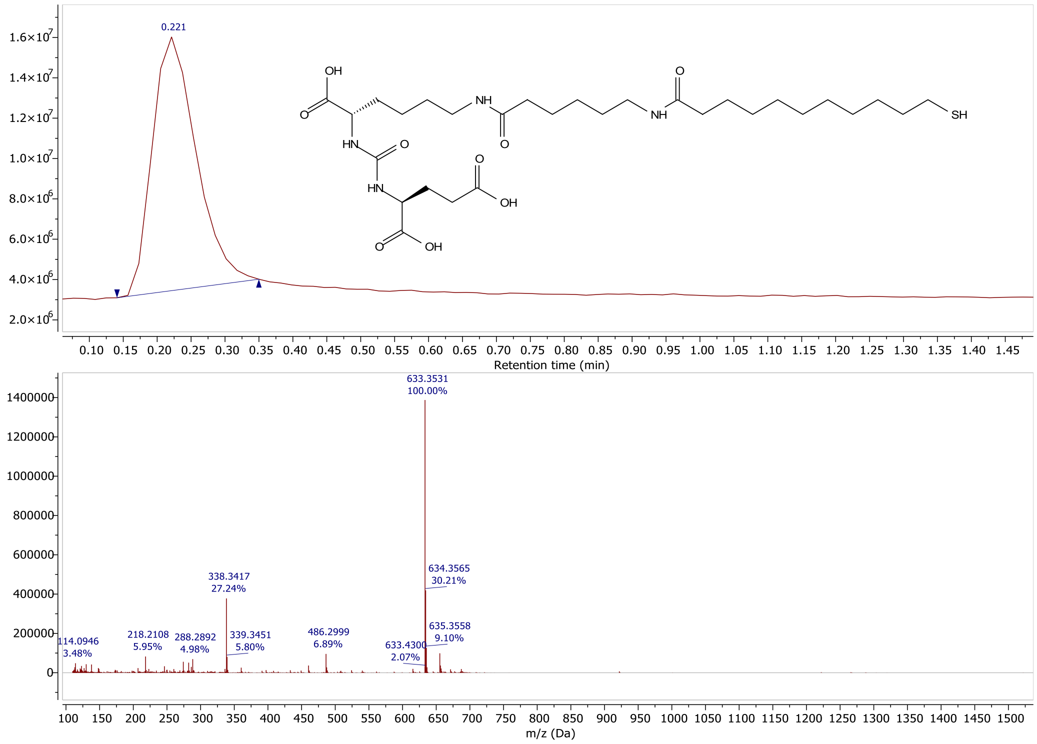

Figure S17. HRMS-ESI of **MUA-AHX-PSMA-I.**

2. Particle synthesis

Gold nanoparticles (AuNPs) were synthesized according to established protocols yielding citrate-stabilized AuNPs with core diameters of d_c ~ 12 nm and dispersities (coefficients of variation) of 5-8 % [5]. The citrate ligands were readily displaced by mixing the AuNP with aqueous or ethanol solutions (100 µl of 1 mM ligand solution per ml of 4-6 nM AuNP solution as synthesized) of the thiolated ligands at room temperature under stirring. After reaction overnight the conjugates were purified and concentrated by repeated centrifugations (30-90 min depending on the volume, 20,000 g). The concentrated AuNP-conjugates could then be resuspended in the desired buffers for stability tests and cell uptake experiments. Figure S18 shows exemplary transmission electron microscopy (TEM) measurements of different batches of AuNPs used in this study, underlining their low dispersity and reproducibility of the mean particle diameter.

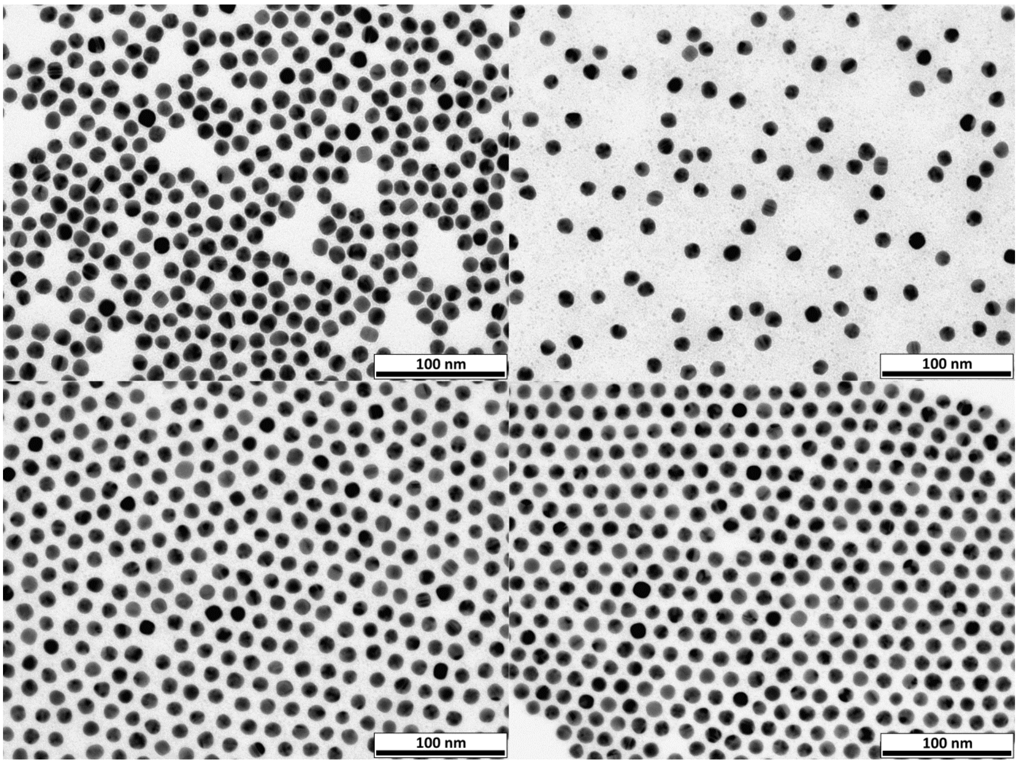

Figure S18. Exemplary TEM measurements of different AuNP batches used in this study. The mean diameter is reproducibly at 12 nm with low dispersities of 5-8 %.

To convert the AuNP concentration c_{NP} to the weight concentration of gold, we assume ideal sphericity of the nanoparticles so the volume of an individual particle with diameter d_c is $V_c = \frac{\pi}{6} d_c^3$. With the density of gold ρ = 19.32 g·cm^{-3} and the number density of particles $N_{NP} = N_A \cdot c_{NP}$ (N_A = Avogadro's number) we obtain the weight concentration of gold $C_{NP} = N \cdot V_c \cdot \rho$ in g·L^{-1} or mg·mL^{-1} respectively. As example, for c_{NP}= 12.5 nM and d_c = 12.0 nm we obtain C_{NP} = 0.13 ± 0.2 mg·mL^{-1}. Note that the dispersity of the AuNPs, even if it is as low as 5% as in this study, affects the calculation of the particle concentration, as well as the calculation of the particle mass, so a direct measurement of the gold weight concentration, as with ICP-MS, is more accurate. We also note that CNP only refers to the mass of the AuNP core, neglecting the mass of the surface coating [6].

We tested several ligand coatings, three coatings based on poly(ethylene glycol) (PEG), which are discussed in the main text, and several coatings without PEG-ligands in follow-up experiments which are discussed in the following. The structures of the ligand shells are summarized in Figures S19-S22. Most functional particles, with and without PEG, are based on the prostate specific membrane antigen inhibitor (Figure S19, PSMA-I), which is the binding motif of the established clinical radiopharmaceutical [^{68}Ga]Ga-PSMA-11 [7–9]. This motif was linked to 11-mercaptoundecanoic

acid (MUA) via an amide bond (custom synthesis, ABX advanced biochemical compounds GmbH, Germany) to yield **MUA-PSMA-I** (Figure S22). The mercaptodecane-spacer was used in all ligands to obtain a high grafting density of the ligands on the AuNP [10,11]. **MUA** coated AuNPs were used as a control (Figure S19). In **MUA-PSMA-I**, the binding motif is located close to the AuNPs' surface (1-2 nm distance) yielding a small conjugate, however its binding ability could be diminished because the size of the conjugate could hamper the insertion of the motif into the binding pocket of the receptor. To account for this, we synthesized a set of additional ligands with an additional 6-aminohexanoic acid (AHX) based spacer: **MUA-AHX-PSMA-I** with the same binding motif and just an additional AHX spacer, **MUA-AHX-GPI** with an alternative binding motif, and **MUA-AHX-Glu** with a terminal glutamic acid as another control with no binding motif (Figure S22).

Another approach allowing even more flexibility of the motif is the use of a longer poly(ethylene glycol)-based spacer [12,13]. To this end we used α-carboxypoly(ethyleneglycol)-ω-(11-mercaptoundecanoic acid) (M = 818 g/mol, **PEGMUA1kCOOH**) (Iris Biotech, Germany, Figure S19), a thiolated PEG-ligand with terminal carboxylic acid groups (-COOH), to coat the AuNPs. The PSMA-I motif was then coupled to the PEG functionalized AuNPs via EDC-coupling (Figure S20). To this end, the terminal carboxylic acid groups were activated by addition of 1-ethyl-3-(3-dimethylaminopropyl)-carbodiimide (EDC) and *N*-hydroxysulfosuccinimide (sulfo-NHS) in ratios of 1:80000:160000 (AuNPs:EDC: sulfoNHS). Samples were purified by centrifugation (twice) after which the particles were redispersed in buffer solution (phosphate buffered saline, PBS, 10 mM, pH 7.6). The reaction with PSMA-I was facilitated by heating up to 70° C for 2 h with a 1000-fold excess of PSMA-I relative to the AuNPs. Non-reacted PSMA-I was removed by centrifugation (thrice) and the particles (**PEG-PSMA-I**, Figure S19) were redispersed in water. The PEG-functionalized particles without coupled PSMA-I were used as a control (**PEGMUA1kCOOH**, Figure S19). As another PEG-control we used particles functionalized with a mixture of a larger PEGMUA-Ligand (M ~ 2kDa, ~ 25 % of the ligand mixture, Figure S19) with no terminal carboxylic acid groups, and 11-mercaptoundecanoic acid (MUA) (M = 218 g/mol, ~ 75 % of the ligand mixture, Figure S19): **PEG-MUA2k/MUA** (Figure S21). Such mixed ligand layers have been demonstrated to allow tuning of the particles' surface charge without compromising particle stability [11]. For particles with 75 % MUA, high unspecific uptake was observed in PC3 cells [11]. These were therefore used as another control of negatively charged PEGylated nanoparticles.

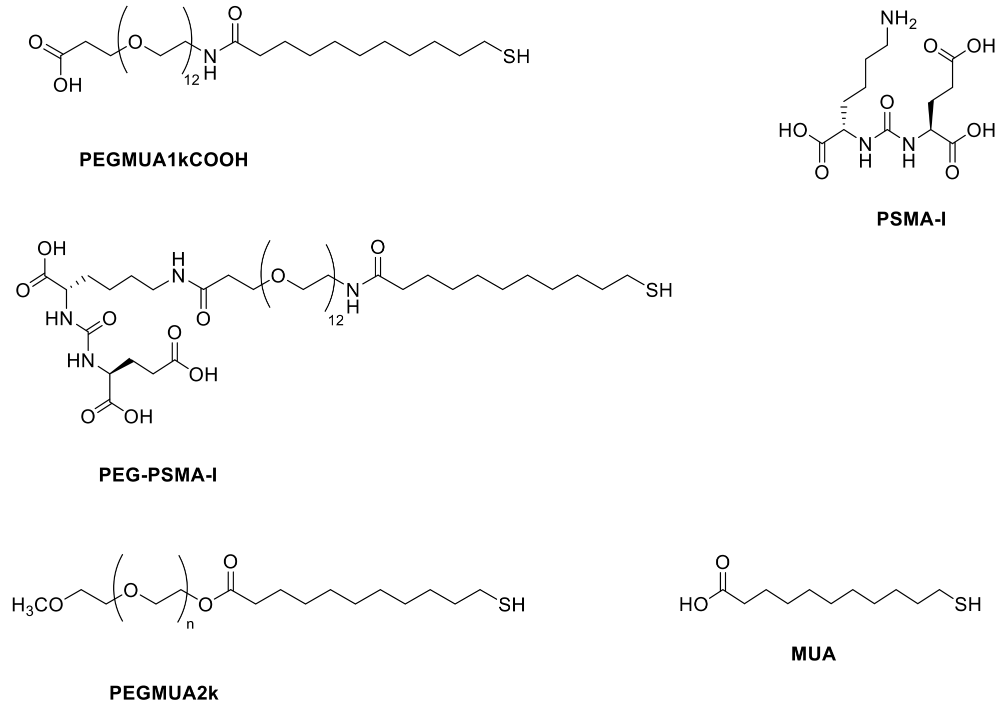

Figure S19. Structures of the ligands discussed in the main text.

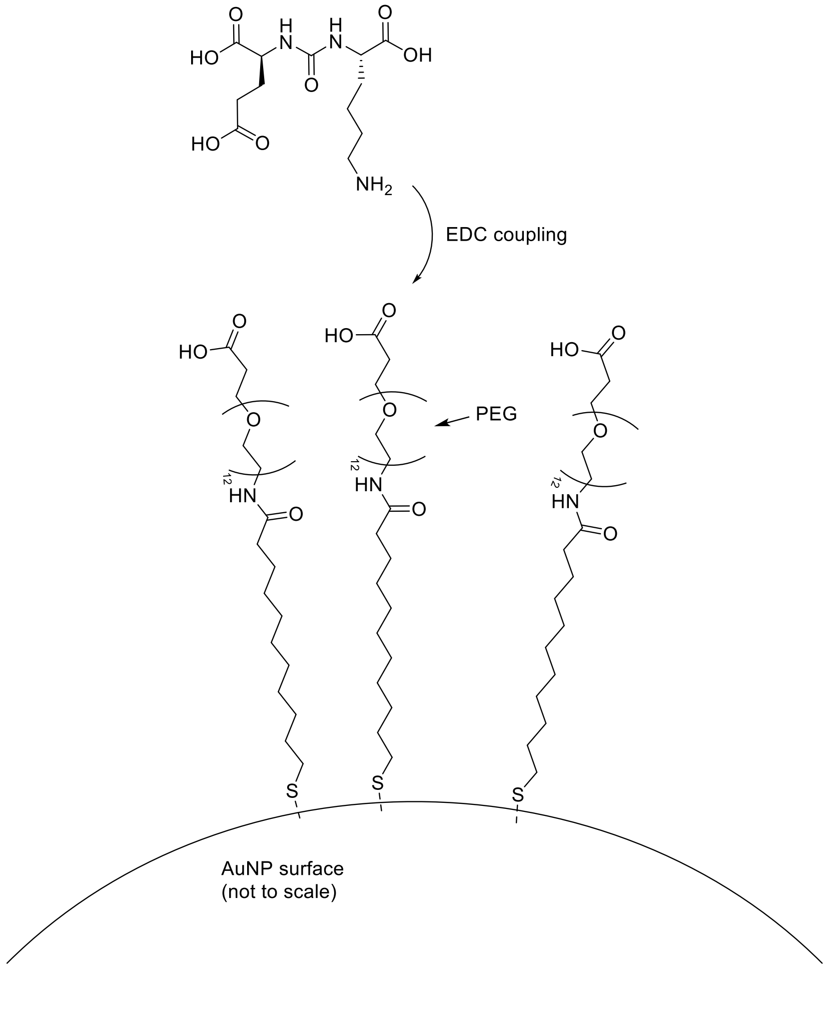

Figure S20. Scheme of the ligand layer PEG-PSMA-I obtained by coupling PSMA-I to surface grafted PEGMUA1kCOOH, resulting in a mixed ligand layer (because not all terminal carboxylic acid groups are coupled to PSMA-I).

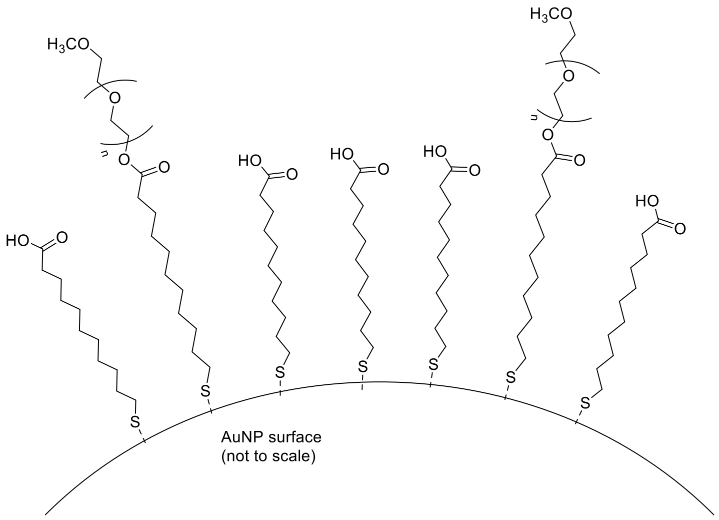

Figure S21. Scheme of the mixed ligand layer consisting of PEGMUA2k (*n* ~ 40) and MUA.

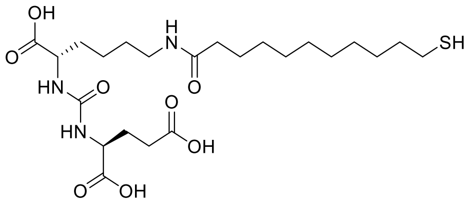

MUA-PMSA-I

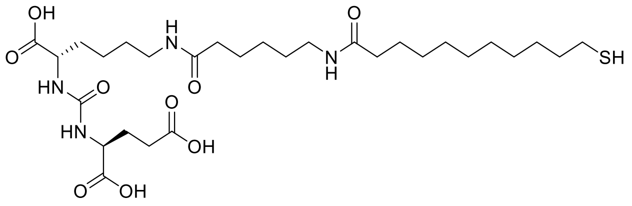

MUA-AHX-PSMA-I

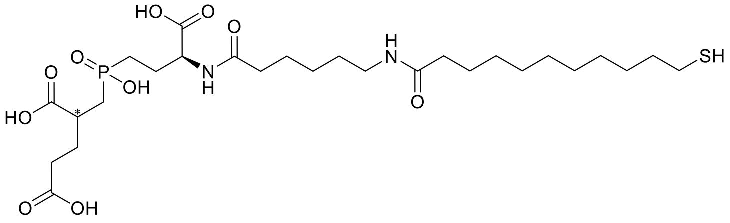

MUA-AHX-GPI

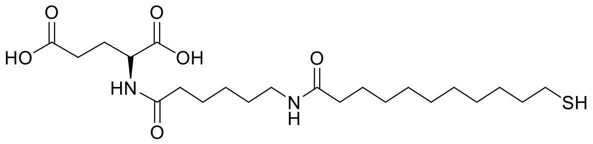

MUA-AHX-Glu

Figure S22. Structures of ligands (without PEG) used in complementary cell experiments.

3. Particle characterization

The stability of the functionalized AuNPs was monitored with dynamic light scattering (DLS) as described [14,6]. Figure S23 shows the number weighted distributions of the hydrodynamic diameters of the samples after preparation including purification and concentration. All samples were colloidally stable in water.

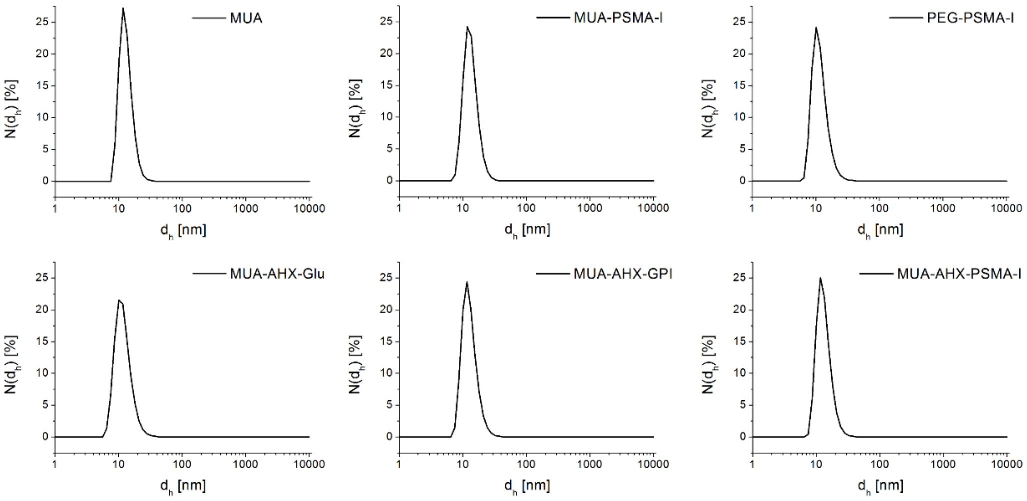

Figure S23. Hydrodynamic diameters of as prepared samples in ultrapure water as indicated. No indication of aggregation was observed.

MUA coated particles were prepared in aqueous solutions with pH 9 to increase the electrostatic colloidal stabilization (by deprotonation of the terminal carboxylic acid groups), because the steric stabilization provided by this small ligand is known to be limited for AuNPs with diameters of d_c ~ 12 nm as used in this study[11]. At lower pH (~5.5 in ultrapure water or ~ 7.4 in PBS) we observed strong indications of agglomeration by DLS and UV/vis absorption spectroscopy. Shift and broadening of the plasmon peak indicate agglomeration of the nanoparticles as well as the shift and limited reproducibility of the apparent hydrodynamic diameter measured by DLS [6,14]. Absorbance spectra of **MUA** in different media at different waiting times are shown in Figure S24.

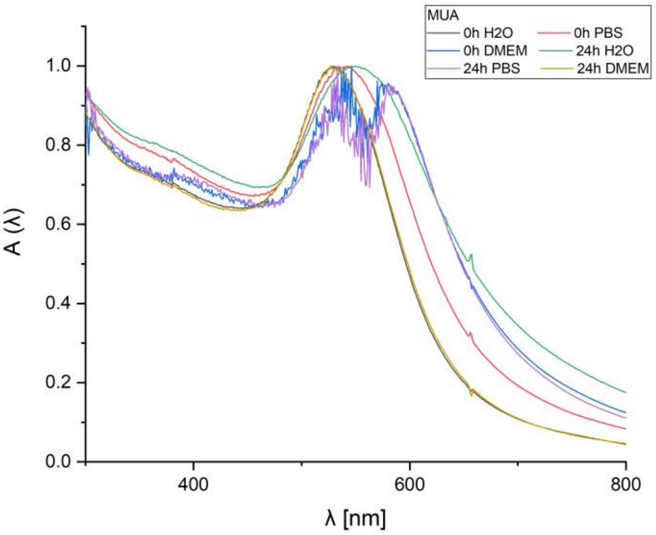

Figure S24. Absorbance spectra of **MUA** dissolved in different media: water (H_2O), PBS, and cell medium (Dulbecco Modified Eagle Medium, DMEM) at different waiting times (0 and 24 h). Broadening of the plasmon peak and scattering effects indicate significant agglomeration of the nanoparticles.

Another destabilization behavior was observed for the sample **MUA-AHX-Glu**. These particles were stable in water as well as in PBS, but in cell medium (DMEM) they agglomerated fast, leading to sedimentation (Figure S25).

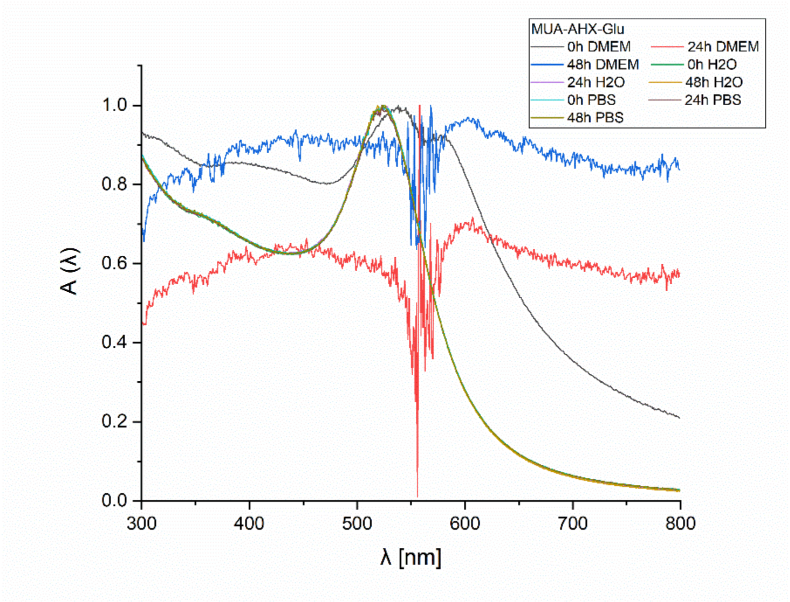

Figure S25. Absorbance spectra of **MUA-AHX-Glu** dissolved in different media: water (H_2O), PBS and cell medium (DMEM) at different waiting times (0 and 24 and 48 h). The particles are well dispersed and colloidally stable in water and PBS, but in cell medium they agglomerate fast and strongly, leading to a turbid and strongly scattering solution.

The other samples exhibited higher stability in water, PBS and cell medium. **MUA-AHX-PSMA-I** were colloidally stable in water, but exhibited notable agglomeration in DMEM (Figure S26).

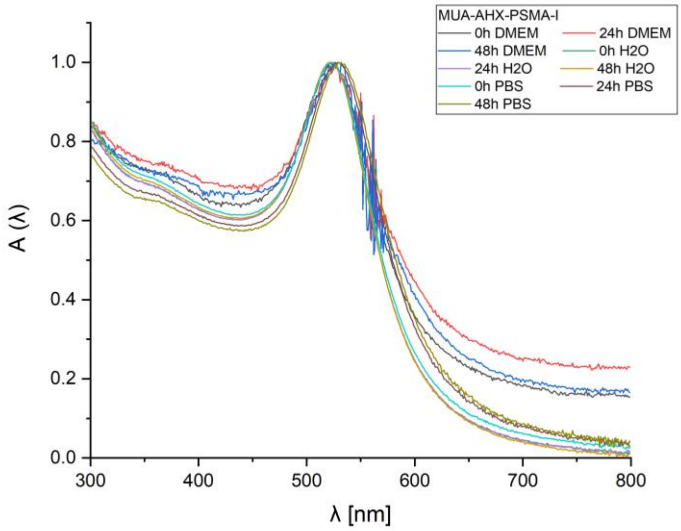

Figure S26. Absorbance spectra of **MUA-AHX-PSMA-I** dissolved in different media: water (H_2O), PBS and cell medium (DMEM) at different waiting times (0 and 24 and 48 h).

MUA-PSMA-I were colloidally more stable in the different media, underlining that a longer ligand does not necessarily provides a better stabilization (Figure S28).

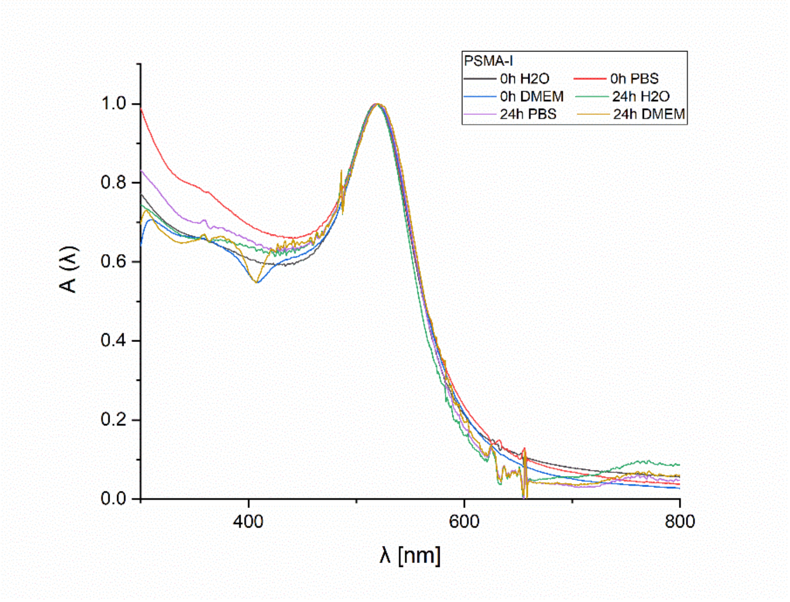

FigureS27. Absorbance spectra of **MUA-PSMA-I** dissolved in different media: water (H_2O), PBS, and cell medium (DMEM) at different waiting times (0 and 24 h).

MUA-AHX-GPI and **PEG-PSMA-I** were also colloidally stable in different media (**Figure S28**) and the stability of PEGylated AuNPs (**PEG-MUA2k/MUA**) in different media was reported previously [11].

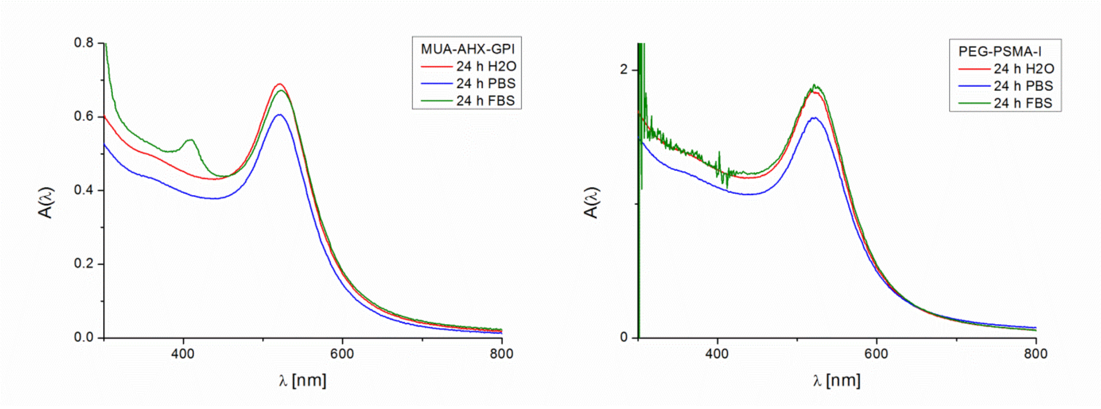

Figure S28. Spectra of **MUA-AHX-GPI** and **PEG-PSMA-I** dissolved in different media: water (H_2O), PBS, and fetal bovine serum (FBS) after 24 h. In PBS a slight decrease of concentration was observed and the cell medium can affect the absorption of the sample but no indication of significant agglomeration was observed.

The characterization of the AuNPs demonstrates that apart from the different ligands on the nanoparticle surface, their major difference is their colloidal stability in cell medium, that can strongly differ, even when the particles are stable in water and PBS.

4. Cell culture and ICP-MS particle uptake protocols

Cell culture experiments were conducted based on protocols described previously [14,15]. PC3-PIP cells with (PC3+PSMA) and PC3 cells without (-PSMA) overexpression of the PSMA receptor were used. PC3+PSMA and PC3-PSMA cells were seeded into 6-well plates at a density of 2×10^5 cells/well in serum containing medium (10% fetal bovine serum, FBS), and were allowed to attach overnight. The next day, the old cell medium was removed and the cells were exposed to 2 mL fresh medium containing the according nanoparticles. The plate was incubated at 37 °C for 24 h or 48 h. After exposure, the nanoparticle solution was removed and cells were washed with 2 mL PBS three times. Then, 0.3 mL trypsin, ethylenediaminetetraacetic acid (EDTA) (0.01% trypsin–EDTA) was added to detach the cells from the plate bottom and transferred to Eppendorf tubes. After centrifugation at 300 rcf for 5 min, cells were resuspended in 1 mL PBS, and 10 μL of this solution was diluted 10 times to count the cell number. Cells were then collected again by centrifugation. For digestion, 75 μL HNO_3 was added and the sample left overnight to lyse the cells, then 150 μL HCl was added to digest the AuNPs. Finally, the samples were further diluted

(1:10) with 2 wt% HCl prior to measuring the elemental concentration of Au in the sample with ICP-MS. The Au-concentrations of all nanoparticle solutions used for uptake experiments were also determined with ICP-MS to calculate the uptake. By dividing the detected mass of elemental gold by the number of cells in the sample, the amount of internalized AuNPs per cell could be given as m_{Au} [pg/cell]. Experiments were performed in independent triplicates, each experiment was with different generations of cells and incubations were done at different days.

ICP-MS determines the amount of Au in a sample solution as ppb (parts per billion), referring to 1 g Au per 10^9 g sample solution. The mass of the sample solution is assumed to be the mass of water only, with a density of 1 g/mL, and thus 1 ppb refers to 10^{-9} g/mL = 1 ng/mL of Au. In a typical sample in the here used protocol there are around 500,000 cells. The cell pellet has around 50 µL volume. In order to digest the cell pellet, 75 µL of HNO_3 is added to lyse the cells overnight. Then 150 µL of HCl is added to form aqua regia to digest the AuNPs. The samples are then diluted 10 times with 2% HCl prior to ICP-MS testing, in order to protect the ICP-MS machine from too high concentrations of acid which could destroy the instrument. The final sample volume thus is 2250 µL, leading to 222 cells/µL ≈ 200,000 cells/mL. A typical result for fully loaded cells was 1000 ppb, which corresponds to 5 pg Au/cell (see Figure S29-S37). In samples without added AuNPs as blank the detected value was around 2 ppb Au, corresponding to 0.01 pg/cell. This is the ICP-MS detection limit in the here used protocol. In other words, the minimum amount of Au in the sample needs to be 0.01 pg/cell · 500,000 cells = 5000 pg. If we consider fully loaded cells with 5 pg/cell, this would correspond to 1000 cells. With our ICP-MS detection protocol we thus would be able to see the minimum amount of 1000 fully loaded cells. This estimation fits well to previous related studies, where with different cells and NPs a detection limit of around 400 cells had been determined[16]. With an autosampler the measurement time per such sample is 150 s.

We note that the numbers given here refer to the used ICP-MS protocol, which was not designed to lead to the minimum possible amount of gold to be detected. IPC-MS can in principle detect Au levels as low as 0.1 µg/L (https://www.eag.com/resources/appnotes/icp-oes-and-icp-ms-detection-limit-guidance/; accessed on 16.3.2021). Taking the here used sample volume of 2250 µL this results in 225 fg ≈ 0.2 pg. This is a much lower value that the 5000 pg as obtained above under different conditions not optimized to determine the minimum amount of Au. The theoretical 0.2 pg ICP-MS limit also can't be directly compared to the 5 pg XFI limit described

in the main article, as also this value depended on the used protocol and thus can't be as good as the theoretical limit.

5. Discussion of ICP-MS uptake results

Here we present the results from the ICP-MS measurement of the cells prepared as detailed in chapter 4 above. First the uptake of **MUA-PSMA-I** and **MUA** is compared (Figure S29-S32).

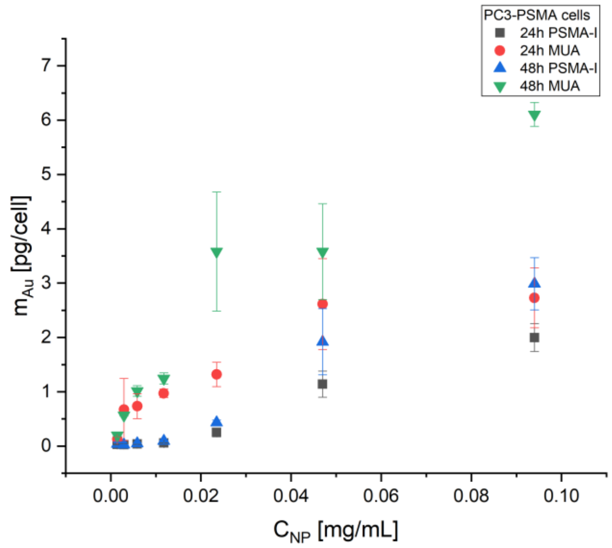

Figure S29. ICP-MS measured uptake of **MUA-PSMA-I** and **MUA** by PC3-PSMA cells (not overexpressing PSMA) after 24 h and 48 h. Amount of internalized AuNPs in terms of mass of gold m_{Au} per cell for different AuNPs and different incubation times. Data are from three independent experiments (n=3) and represent mean values ± standard deviations.

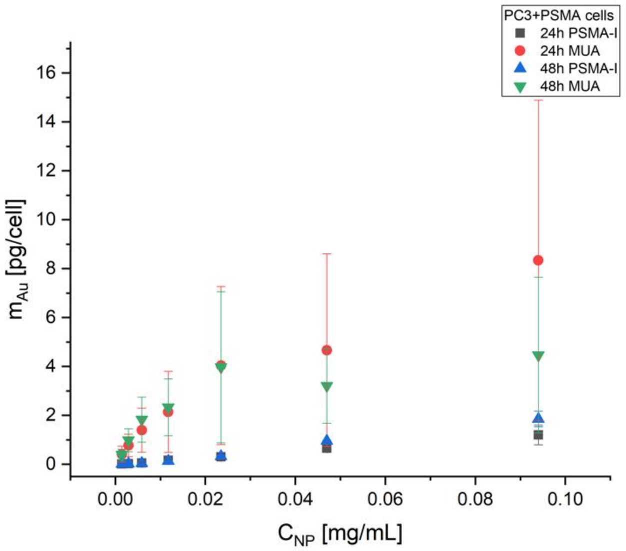

Figure S30. ICP-MS measured uptake of **MUA-PSMA-I** and **MUA** by PC3+PSMA cells (overexpressing PSMA) after 24 h and 48 h. Amount of internalized AuNPs in terms of mass of gold m_{Au} per cell for different AuNPs and different incubation times. Data are from three independent experiments (n=3) and represent mean values ± standard deviations.

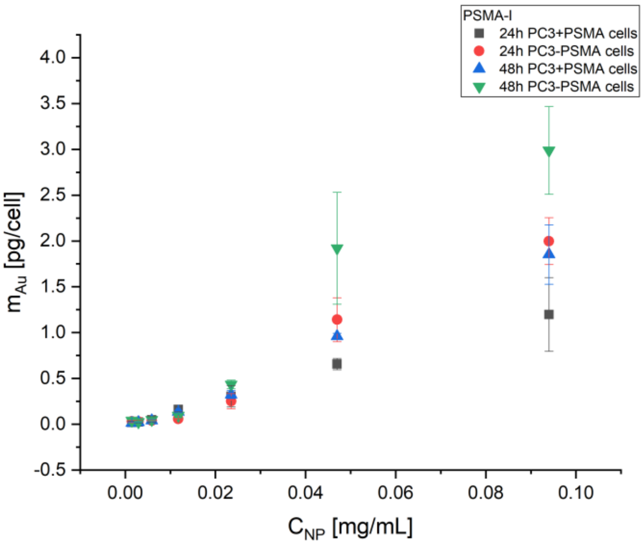

Figure S31. ICP-MS measured uptake of **MUA-PSMA-I** by PC3+PSMA and PC3-PSMA cells after 24 h and 48 h. Same data as in Figure S27 and S28 but plotted for the different types of cells investigated here.

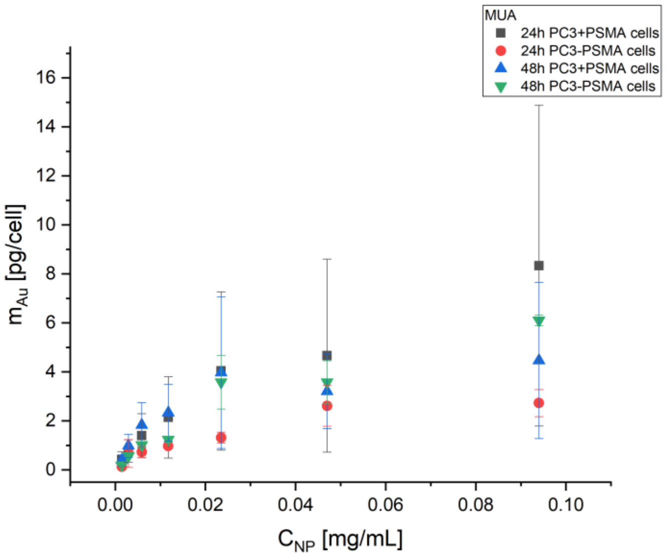

Figure S32. ICP-MS measured uptake of **MUA** by PC3+PSMA and PC3-PSMA cells after 24 h and 48 h. Same data as in Figure S29 and S30 but plotted for the different types of cells investigated here.

It was observed, that there was higher uptake of **MUA** than for **MUA-PSMA-I** for both cell lines. This shows that uptake is dominated not by specific targeting, but by colloidal stability, whereby colloidally less stable AuNPs sediment on top of the cells and thus are incorporated to a higher extent [14,17]. Also, the uptake of **MUA-PSMA-I** is higher in PC3-PSMA cells not overexpressing the PSMA receptor, indicating cell lineage effects.

Figure S33 and S34 summarize the results of uptake experiments with **MUA-AHX-PSMA-I**, **MUA-AHX-GPI** and **MUA-AHX-Glu**.

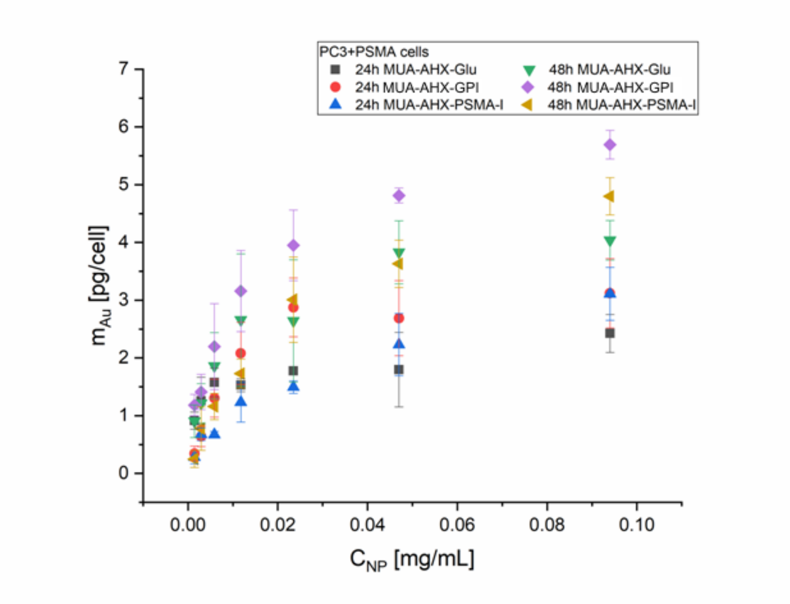

Figure S33. ICP-MS measured uptake of **MUA-AHX-PSMA-I, MUA-AHX-GPI and MUA-AHX-Glu** by PC3+PSMA cells (overexpressing PSMA) after 24 h and 48 h. Amount of internalized AuNPs in terms of mass of gold m_{Au} per cell for different AuNPs and different incubation times. Data are from three independent experiments (n=3) and represent mean values ± standard deviations.

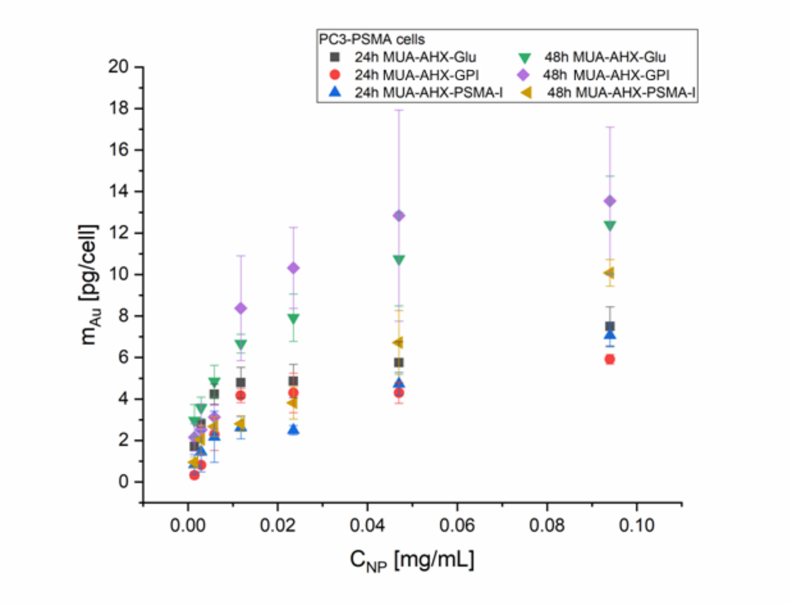

Figure S34. ICP-MS measured uptake of **MUA-AHX-PSMA-I, MUA-AHX-GPI and MUA-AHX-Glu** by PC3-PSMA cells (not overexpressing PSMA) after 24 h and 48 h. Amount of internalized AuNPs in terms of mass of gold m_{Au} per cell for different AuNPs and different incubation times. Data are from three independent experiments (n=3) and represent mean values ± standard deviations. Note the different y-scale compared to Figure S33.

For all cells and nanoparticles the typical concentration dependent endocytosis was seen, as shown in Figures S33 and S34. The highest uptake is observed for **MUA-AHX-GPI**, however the uptake was higher in PC3-PSMA cells not overexpressing the PSMA receptor. This was also the case for the other particles. To facilitate comparison, the uptake for each of the different nanoparticles is plotted for the two different cell types in Figures S35-S37.

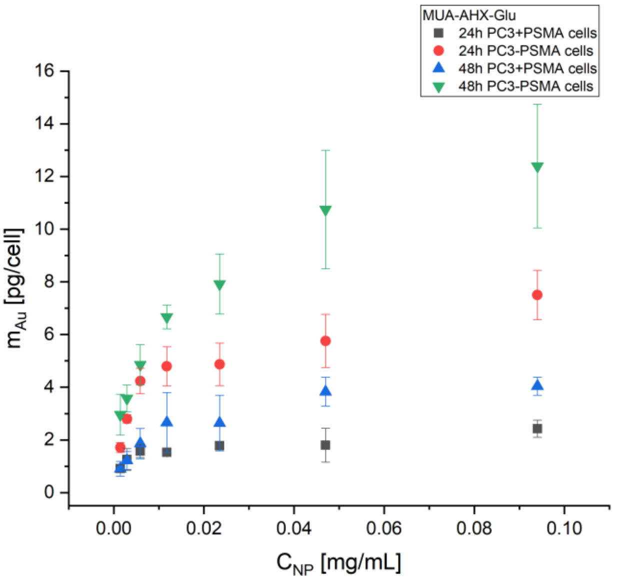

Figure S35. ICP-MS measured uptake of **MUA-AHX-Glu** by PC3+PSMA and PC3-PSMA cells after 24 h and 48 h. Same data as in Figures S33 and S34 but plotted for the different types of cells investigated here.

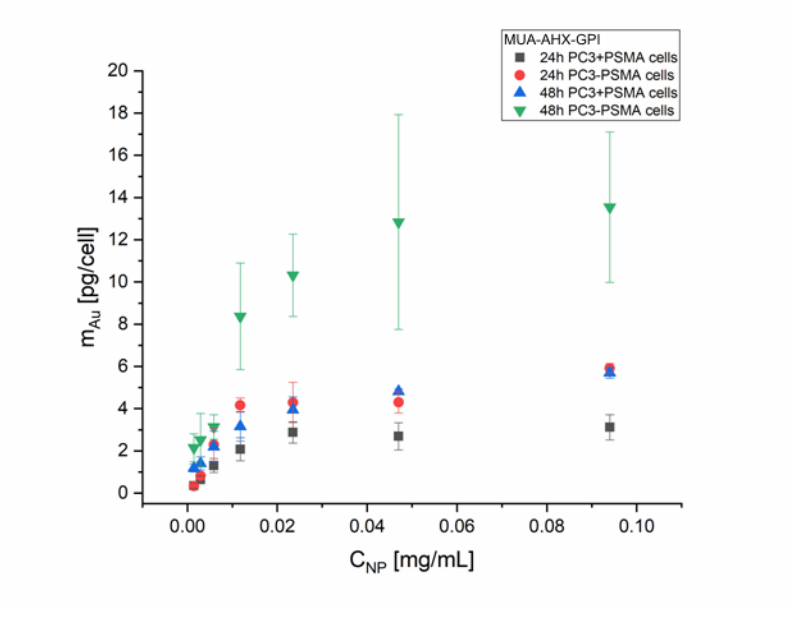

Figure S36. ICP-MS measured uptake of **MUA-AHX-GPI** by PC3+PSMA and PC3-PSMA cells after 24 h and 48 h. Same data as in Figures S33 and S34 but plotted for the different types of cells investigated here.

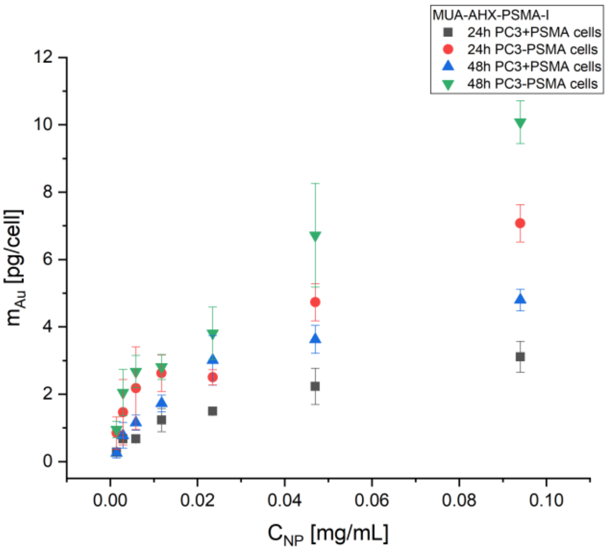

Figure S37. ICP-MS measured uptake of **MUA-AHX-PSMA-I** by PC3+PSMA and PC3-PSMA cells after 24 h and 48 h. Same data as in Figures S33 and S34 but plotted for the different types of cells investigated here.

In summary in the data presented here, there is no indication that uptake is specific due to binding of PMSA binding ligands to PSMA receptor modified cells. Colloidal stability and cell linage effect and thus non-specific effects have determined uptake in the 2 data sets.

The PEGylated samples (**PEG1kCOOH**, **PEG-PSMA-I** and **PEG-MUA2k/MUA**) discussed in the main text are colloidally more stable, however the highest uptake was found for **PEGMUA2k/MUA**, i.e. nanoparticles with no PSMA binding ligand. Thus, the capability of XFI for measuring low gold concentrations in cells is demonstrated, but conclusions regarding specific uptake cannot be drawn at this point and thus are not discussed in the main article.

The ICP-MS results shown here indicate a saturation level of cells with AuNPs at around 5 pg/cell. In case of the XFI measurements in the main paper the maximum amount of AuNPs per cell was around 400 pg per 888 cells (cf. Figure 1) ≈ 0.45 pg/cell, which is one order of magnitude lower

than the ICP-MS data. However, ICP-MS and XFI recordings were not done under the same conditions. First, the surface capping of the NPs was different. For XFI the incubation time was 16 h. ICP-MS was recorded after 24 h and 48 h incubation time and the data show that uptake was not saturated yet at 24 h. For XFI an incubation concentration of C_{NP} = 0.13 mg/mL was used, which is higher than the maximum concentration of $C_{NP} \approx 0.095$ mg/mL as used for ICP-MS. Higher NP concentrations may impair cell viability. In addition, some of the NPs may only be attached to the outer cell membrane instead of being endocytosed [18]. These NPs would be wrongfully counted by ICP-MS as internalized NPs, but upon the gel embedding procedure used for XFI measurements might be detached from cells. In addition, some NPs may have been lost during storage time of the agarose-embedded cells before actual XFI measurements. Thus, there is a range of possible explanations to account for the different determined amount of Au per cell for the separate ICP-MS and XFI studies shown here

6. XFI significances and fit values

In order to underline the extrapolation given in the main text from the measured data to the optimized sensitivity limit of our XFI approach, Figure S38 below shows the statistical significance and chi-squared values for the measured data given in Fig. 1 in the main text. One could thus directly scale down the measured AuNP-mass to such values that correspond to a Z=3 level, which is already around the order of magnitude reached by the optimization of XFI towards the shown sensitivity limit. As of note, the detectable AuNP-mass scales directly with Z. Hence, if Z = 100, one could measure 33-times less AuNPs, reaching the statistical limit of Z = 3, without any optimization. If one applies, in addition, the optimization as discussed in the main text, one can reach the level of around 5 pg AuNP mass in the X-ray beam volume.

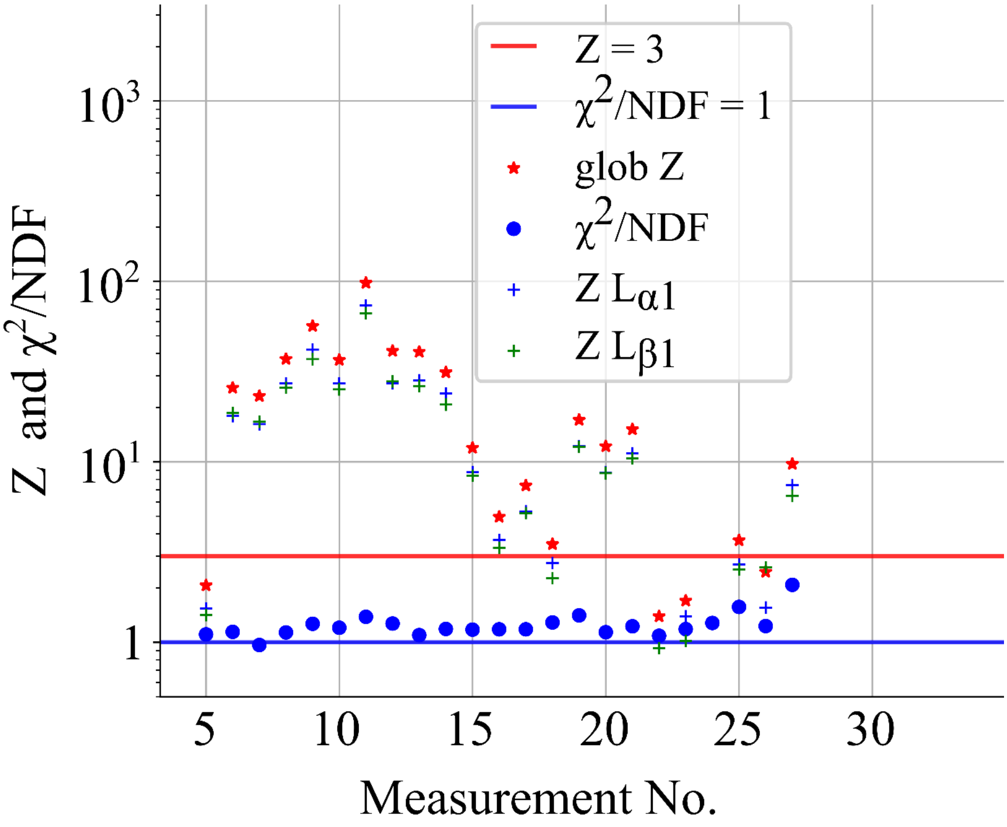

Figure S38. Significance and X^2/ndf fit values for measurements shown in Figure 1. The detection limit of Z=3 is marked with a red line, while the ideal X^2/ndf=1 value is shown with a blue line. Significances are shown for the L_α and the L_β region as well as the global Z value, L_α and L_β combined.

7. References

1. D. Marchand, J. Martinez, and F. Cavelier, "ChemInform Abstract: Straightforward Synthesis of Chiral Silylated Amino Acids Through Hydrosilylation," *Cheminform*, vol. 39, 2008, doi: 10.1002/chin.200842190.
2. D. Bartley and J. Coward, "A Stereoselective Synthesis of Phosphinic Acid Phosphapeptides Corresponding to Glutamyl-γ-glutamate and Incorporation into Potent Inhibitors of Folylpoly-γ-glutamyl Synthetase," *The Journal of organic chemistry*, vol. 70, pp. 6757–74, 2005, doi: 10.1021/jo0507439.
3. C. Camodeca *et al.*, "Synthesis and in vitro Evaluation of ADAM10 and ADAM17 Highly Selective Bioimaging Probes," *ChemMedChem*, vol. 13, 2018, doi: 10.1002/cmdc.201800482.
4. M. Benešová *et al.*, "Linker Modification Strategies To Control the Prostate-Specific Membrane Antigen (PSMA)-Targeting and Pharmacokinetic Properties of DOTA-Conjugated PSMA Inhibitors," *Journal of Medicinal Chemistry*, vol. 59, 2016, doi: 10.1021/acs.jmedchem.5b01210.
5. F. Schulz, T. Homolka, N. G. Bastus, V. Puntes, H. Weller, and T. Vossmeyer, "Little Adjustments Significantly Improve the Turkevich Synthesis of Gold Nanoparticles," *Langmuir*, vol. 30, pp. 10779–10784, 2014.
6. J. Hühn *et al.*, "Selected Standard Protocols for the Synthesis, Phase Transfer, and Characterization of Inorganic Colloidal Nanoparticles," *Chemistry of Materials*, vol. 29, 2016, doi: 10.1021/acs.chemmater.6b04738.
7. K. Rahbar, A. Afshar-Oromieh, H. Jadvar, and H. Ahmadzadehfar, "PSMA Theranostics: Current Status and Future Directions," *Molecular Imaging*, vol. 17, p. 1536012118776068, 2018, doi: 10.1177/1536012118776068.
8. S. R. Banerjee *et al.*, "68Ga-Labeled Inhibitors of Prostate-Specific Membrane Antigen (PSMA) for Imaging Prostate Cancer," *Journal of Medicinal Chemistry*, vol. 53, no. 14, pp. 5333–5341, 2010, doi: 10.1021/jm100623e.
9. M. Eder *et al.*, "68Ga-Complex Lipophilicity and the Targeting Property of a Urea-Based PSMA Inhibitor for PET Imaging," *Bioconjugate Chemistry*, vol. 23, no. 4, pp. 688–697, 2012, doi: 10.1021/bc200279b.
10. F. Schulz *et al.*, "Structure and Stability of PEG- and Mixed PEG-Layer-Coated Nanoparticles at High Particle Concentrations Studied In Situ by Small-Angle X-Ray Scattering," *Particle & Particle Systems Characterization*, vol. 35, p. 1700319, 2017, doi: 10.1002/ppsc.201700319.
11. F. Schulz *et al.*, "Ligand Layer Engineering To Control Stability and Interfacial Properties of Nanoparticles," *Langmuir : the ACS journal of surfaces and colloids*, vol. 32, 2016, doi: 10.1021/acs.langmuir.6b01704.
12. D. Luo, X. Wang, S. Zeng, G. Ramamurthy, C. Burda, and J. Basilion, "Prostate-specific membrane antigen targeted gold nanoparticles for prostate cancer radiotherapy: does size matter for targeted particles?," *Chemical Science*, vol. 10, 2019, doi: 10.1039/C9SC02290B.
13. J. Mangadlao *et al.*, "Prostate Specific Membrane Antigen Targeted Gold Nanoparticles for Theranostics of Prostate Cancer," *ACS Nano*, vol. 12, 2018, doi: 10.1021/acsnano.8b00940.
14. M. Xu *et al.*, "How Entanglement of Different Physicochemical Properties Complicates the Prediction of in Vitro and in Vivo Interactions of Gold Nanoparticles," *ACS Nano*, vol. 12, 2018, doi: 10.1021/acsnano.8b04906.
15. Z. Liu *et al.*, "Biodegradation of Bi-Labeled Polymer-Coated Rare-Earth Nanoparticles in Adherent Cell Cultures," *Chemistry of Materials*, vol. XXXX, 2019, doi: 10.1021/acs.chemmater.9b03673.
16. P. Nold *et al.*, "Optimizing conditions for labeling of mesenchymal stromal cells (MSCs) with gold nanoparticles: A prerequisite for in vivo tracking of MSCs," *Journal of Nanobiotechnology*, vol. 15, 2017, doi: 10.1186/s12951-017-0258-5.
17. N. Feliu Torres, X. Sun, R. Alvarez-Puebla, and W. Parak, "Quantitative particle-cell interaction - about some basic physicochemical pitfalls," *Langmuir : the ACS journal of surfaces and colloids*, vol. 33, p. 6639–6646, 2017, doi: 10.1021/acs.langmuir.6b04629.
18. M. Semmling, O. Kreft, A. M. Javier, G. Sukhorukov, J. Käs, and W. Parak, "A novel flow-cytometry-based assay for cellular uptake studies of polyelectrolyte microcapsules.," *Small*, vol. 4 10, pp. 1763–8, 2008.
